# Bilayer Permeabilization and the ‘Sand-in-a-Gearbox’ Mechanism for Membrane-Active Molecules: Which Is Which?

**DOI:** 10.3390/antibiotics15080741

**Published:** 2026-07-31

**Authors:** Zahra Vaezi, Annalisa Bortolotti, Cristiano Di Stefano, Simone Bonacorsi, Valerio Santucci, Federico Carneri, Christopher Aisenbrey, Mohini M. Konai, Yash Acharya, Mariano Venanzi, Roya Khosravi-Far, Burkhard Bechinger, Jayanta Haldar, Lorenzo Stella, Daniela Roversi, Gianfranco Bocchinfuso

**Affiliations:** 1 Department of Chemical Sciences and Technologies, Tor Vergata University of Rome, Via della Ricerca Scientifica 1, 00133 Rome, Italy; zahra.vaezi@modares.ac.ir (Z.V.); bortolottiannalisa@libero.it (A.B.); cristianodst633@gmail.com (C.D.S.); simone.bonacorsi92@gmail.com (S.B.); valerio135v@gmail.com (V.S.); federico.carneri@uniroma2.it (F.C.); venanzi@uniroma2.it (M.V.); stella@stc.uniroma2.it (L.S.); 2 Department of Biomaterial, Faculty of Interdisciplinary Science and Technologies, Tarbiat Modares University, Tehran 14115-154, Iran; 3 Department of Chemistry, Biology and Biotechnology, Photoinduced Processes and Technologies Doctoral School, Perugia University, 06123 Perugia, Italy; 4 RMN et Biophysique des Membranes, Institut de Chimie de Strasbourg, UMR 7177, Université de Strasbourg, 4, Rue Blaise Pascal, 67000 Strasbourg, France; aisenbrey@unistra.fr (C.A.); bechinge@unistra.fr (B.B.); 5 Jawaharlal Nehru Centre for Advanced Scientific Research, Bengaluru 560064, India; mmkonai@tezu.ernet.in (M.M.K.); yash@jncasr.ac.in (Y.A.); jayanta@jncasr.ac.in (J.H.); 6 Medicinal Chemistry and Biomaterial Laboratory (MCBL), Department of Chemical Sciences, Tezpur University, Napaam, Tezpur 784028, Assam, India; 7 InnoTech Precision Medicine, Boston, MA 02142, USA; roya@innotechprecisionmed.com; 8 Institut Universitaire de France, 75005 Paris, France

**Keywords:** antimicrobial peptides, membrane dynamics, fluorescence spectroscopy, peptidomimetics

## Abstract

**Background/Objectives:** Antimicrobial peptides are promising agents for combating resistant infections. They exhibit bactericidal activity against a wide range of microbes, primarily by disrupting the permeability of the bacterial membrane and ultimately causing cell death. Effective bacterial killing requires a high number of membrane-bound peptide molecules. Therefore, it is conceivable that peptide accumulation on the membrane could also interfere with essential cellular processes by altering bilayer dynamics, a hypothesis referred to as the “sand-in-a-gearbox” model. **Methods:** We systematically investigated how membrane dynamics is affected by a set of well-characterized yet highly diverse peptides: the natural AMP magainin 2, the toxin melittin, the synthetic peptides LAH4 and Killer-FLIP, and small membrane-active peptidomimetics with bactericidal activity. These effects were examined using fluorescence spectroscopy techniques, by measuring anisotropy, generalized polarization, and excimer formation of specific probes inserted at different depths within the lipid bilayer. **Results:** The activity of all compounds extends beyond membrane permeabilization, and the perturbation of membrane dynamics, often associated with the so-called “sand-in-a-gearbox” mechanism, is a common feature among all systems analyzed. The membrane-active compounds induced a stiffening of the phospholipid bilayer by reducing lipid lateral mobility and decreasing water penetration, at least on the nanosecond timescale accessible to fluorescence measurements. **Conclusions:** The concentration range in which this behavior occurred was the same for all compounds studied. This threshold is, generally, higher than that required for membrane permeabilization and reflects near-complete coverage of the bilayer surface.

## 1. Introduction

The emergence and global dissemination of drug-resistant pathogens have elevated antimicrobial resistance (AMR) to one of the most pressing threats to public health worldwide (https://data.who.int/dashboards/amr/overview, accessed on 11 February 2026). Antimicrobial peptides (AMPs) possess several remarkable features that make them highly promising candidates to combat the global spread of antibiotic-resistant “superbugs”. These include their rapid killing of pathogens, their generally nonspecific mechanism of action involving membrane permeabilization, which makes the development of resistance highly unlikely, and, generally, their inherent selectivity toward microbial cells [1,2].

AMPs are extremely heterogeneous in terms of secondary structure, length, and amino acid composition, and no conserved sequence motif can be identified. In the present work, we focus on linear, cationic antimicrobial peptides that share several common physicochemical features. AMPs of this family consist of relatively short peptide sequences (typically 10–50 amino acids) enriched in basic and hydrophobic residues. Although diverse secondary structures have been reported, they are generally unstructured in aqueous solution and often fold into α-helical conformations upon interaction with phospholipid membranes [3,4,5,6], conferring an amphipathic character. The presence of basic amino acids confers an overall positive charge on AMPs, which plays an essential role in driving the initial electrostatic attraction to negatively charged bacterial membranes and contributes to their selectivity for microbial cells over zwitterionic mammalian cells [2].

It is widely accepted that the primary antimicrobial mechanism of cationic AMPs involves perturbation of the bacterial membrane, leading to dissipation of electrochemical potential, disruption of lipid asymmetry, and leakage of essential metabolites and cellular components. However, additional mechanisms may contribute to bacterial killing. Increasing evidence suggests that AMPs can also modulate the host immune response by activating leukocytes, enhancing the recruitment of immune cells (such as neutrophils, monocytes, and T lymphocytes) to the site of infection, and regulating processes including angiogenesis, apoptosis, and wound healing [7,8,9]. A hypothesis proposed by Sahl and co-workers, known as the “sand-in-the-gearbox” model [10], suggests that, rather than killing bacteria solely through membrane permeabilization, AMPs may interfere with the coordinated, highly dynamic function of membrane-bound multienzyme complexes. The centrality of the bacterial membrane to various physiological and metabolic functions of the bacterial cell, and the significantly detrimental effects that AMPs have on the structural and functional integrity of the bacterial membrane, further support the possibility that AMPs may act through such a mechanism.

Previously, we demonstrated that killing requires complete peptide coverage of the cell surface in both Gram-negative and Gram-positive bacteria [11,12]. In parallel, using fluorescence spectroscopy, we recently showed that the activity of two natural AMPs, alamethicin and PMAP-23, extends beyond membrane permeabilization and also involves modifications in bilayer dynamics, leading to decreased water penetration and lipid lateral diffusion. Remarkably, in the same work, these effects are not restricted to model vesicles but also occur in live bacterial cells [13].

Beyond the description of the behavior of specific AMPs, due to the intrinsic diversity of this class of molecules, it is of pivotal importance to understand whether, or to what extent, this behavior can be considered a general feature of AMPs and AMP-mimicking compounds. To this end, in this study, we systematically investigated the effects of several membrane-active compounds on membrane dynamics.

First, we chose three well-characterized yet structurally diverse antimicrobial peptides: the natural AMP magainin 2, the toxin melittin, and the synthetic peptide LAH4. Magainin 2 was among the first AMPs to be identified. It is a 23-residue cationic peptide isolated from the skin of the African frog Xenopus laevis, exhibiting strong antimicrobial and antitumor activity [14,15,16]. It permeabilizes negatively charged membranes by forming an amphipathic helix and creates membrane defects via a “carpet/toroidal” mechanism [17].

Melittin is a 26-residue, highly amphiphilic peptide isolated from the venom of the European honeybee Apis mellifera [18,19,20]. Biophysical studies indicate that this peptide exerts its antimicrobial activity via the “carpet” mechanism [21,22]. However, despite its potent antibacterial activity, melittin is also notorious for its toxicity toward eukaryotic cells, thereby limiting its clinical application as an antimicrobial agent [23]. Both magainin 2 and melittin have been intensively studied for their interaction with the membrane models; different reviews are available, which summarize these studies [24].

LAH4 is a synthetic peptide with both membrane permeabilization and cell-penetrating abilities. Composed of leucines, alanines, four histidines, and terminal lysine pairs, it can exhibit different charge states and membrane orientations in response to changes in pH [25,26]. Its helix lies parallel to membranes at acidic pH and becomes transmembrane at neutral or basic pH [25,27]. The peptide’s orientation is also affected by factors such as counter-ions and lipid composition [28]. Notably, a study found that in phosphatidylglycerol bilayers, its surface alignment is stabilized [29].

We extended this investigation to other membrane-active compounds, including the peptide Killer-FLIP [30], which exhibits remarkably potent cytotoxicity across a variety of cancer cell lines and inhibits tumor growth *in vivo*. The peptide was originally designed to interfere with apoptosis signaling [31], but it was subsequently shown to act independently of apoptosis and to be accompanied by loss of membrane integrity [31]. Table 1 summarizes the investigated peptides.

**Figure 1:**
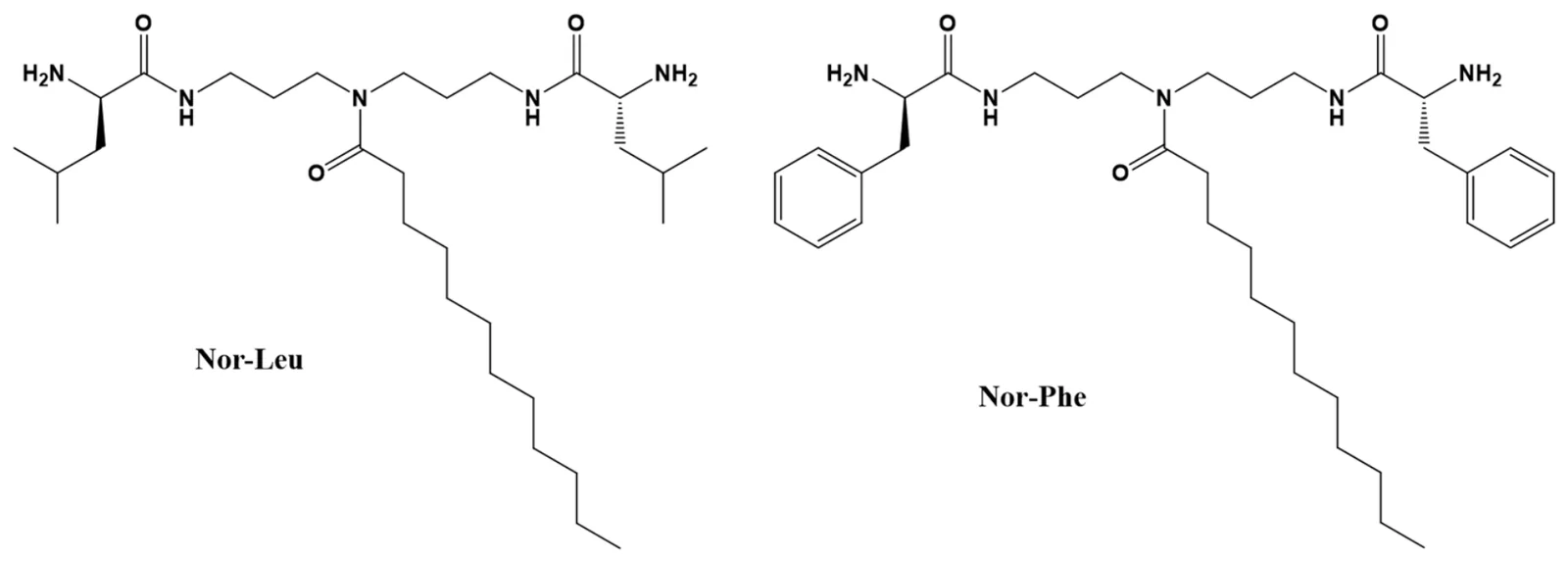
Chemical structure of the peptidomimetics Nor-Leu and Nor-Phe.

Increasing research efforts are focused on developing synthetic molecules that emulate both the antibacterial activity and the underlying mechanisms of AMPs while circumventing some of their intrinsic limitations. Among these, we focused our attention on a class of peptidomimetics, for which it has been demonstrated that they act by disrupting the bacterial membranes, featuring positive charges and a pendant aliphatic group around a norspermidine residue (Figure 1) [32,33]. These compounds demonstrate strong activity against both Gram-positive and Gram-negative bacteria, including drug-resistant strains, with MIC values in the micromolar range [34]. While these molecules embody some of the physicochemical characteristics of AMPs, such as cationic charge and hydrophobicity, they differ significantly in terms of size, molecular weight and nature of chemical backbone. In this case, we aim to determine whether, from this perspective, the features of these molecules overlap those of the AMPs. The effects of all membrane-active compounds were investigated using fluorescence spectroscopy, a powerful experimental technique that allows the monitoring of dynamic phenomena on the nanosecond timescale.

## 2. Results

### 2.1. Perturbation of Membrane Permeability

Beyond their affinity for lipid bilayers, the activity of membrane-permeabilizing molecules mainly depends on their ability to insert and destabilize the bilayer [34]. To determine the range of peptide concentrations that induces membrane permeability and to relate this to other possible membrane effects, we measured peptide-induced membrane leakage. The permeability of the bilayer induced by melittin and magainin 2 was studied by entrapping the fluorescent probe carboxyfluorescein (CF) inside lipid vesicles at a concentration high enough to induce quenching of the dye by self-association [35].

A fixed amount of vesicles (lipid concentration 50 µM) was titrated with increasing concentrations of peptide, and the release kinetics of the probe were recorded (Figure S5).

We tested LAH4 activity at both pH 7.4 and 5.0 to study the effect of peptide charge on its behavior by entrapping calcein, rather than CF, inside the lipid vesicles, because at acidic pH the latter fluorophore is spontaneously released very quickly [36], while calcein does not suffer from this limitation [37]. We verified that peptide-induced leakage at pH 7.4 is not significantly affected by the choice of the particular dye used (Figure S1).

**Figure 2:**
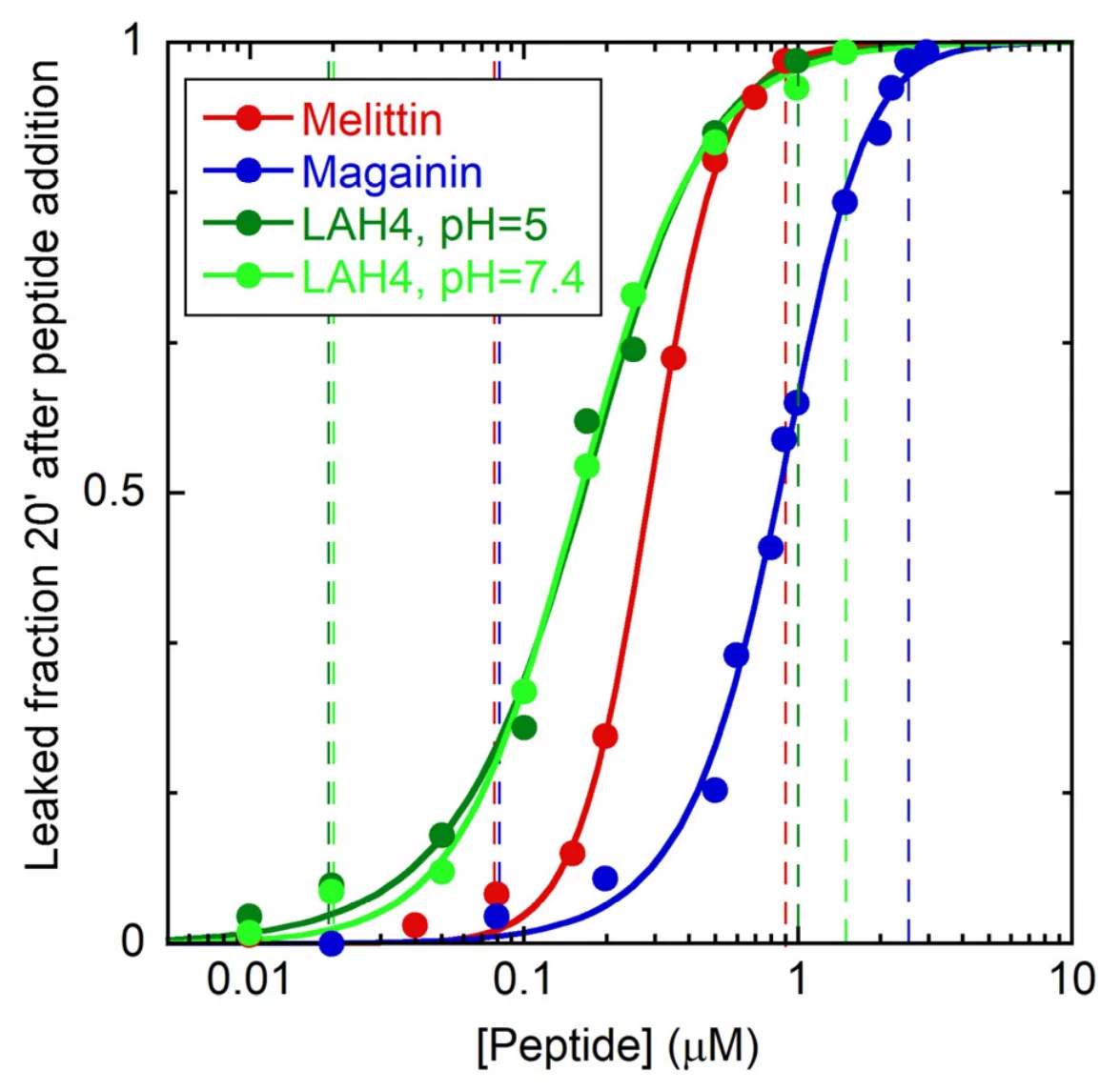
Dye leakage from POPC/POPG (2:1 molar ratio) vesicles (lipid concentration 50 µM) induced by magainin 2, melittin, and LAH4 at 298 K, in phosphate buffer 10 mM, and at pH 7.4, unless stated otherwise. The vertical dashed lines show the peptide concentration range that induced membrane leakage from >3% to >97%.

The leakage assay provided that LAH4 exhibits the highest membrane-perturbation activity, followed by melittin and magainin 2, whose bilayer perturbation abilities are reduced by a factor of ten relative to LAH4 (Figure 2). In addition, the membrane-perturbing behavior of LAH4 is the same at both pHs as previously reported [37]; the same paper highlighted that the affinity for negatively charged membranes is slightly perturbed. Therefore, we can speculate that peptide activity is not influenced by its orientation on the membrane, at least in POPC/POPG liposomes [27,29,37,38]. This allows one to evaluate the peptide-induced effects on the bilayer without any additional complication caused by possible variation in peptide orientation or membrane permeabilization activity.

### 2.2. Perturbation of Membrane Fluidity and Order: Steady-State Anisotropy

The effects of the three peptides on membrane fluidity (local lipid viscosity and packing of membrane) were assessed by steady-state fluorescence anisotropy. This spectroscopic technique allows the determination of the speed and amplitude of a fluorophore’s rotational motion during its excited-state lifetime. Slow (compared to the lifetime) and/or limited motions correspond to high anisotropy values (with the maximum, limiting value being 0.4), while fast and wide rotations yield anisotropy values close to zero [39].

**Figure 3:**
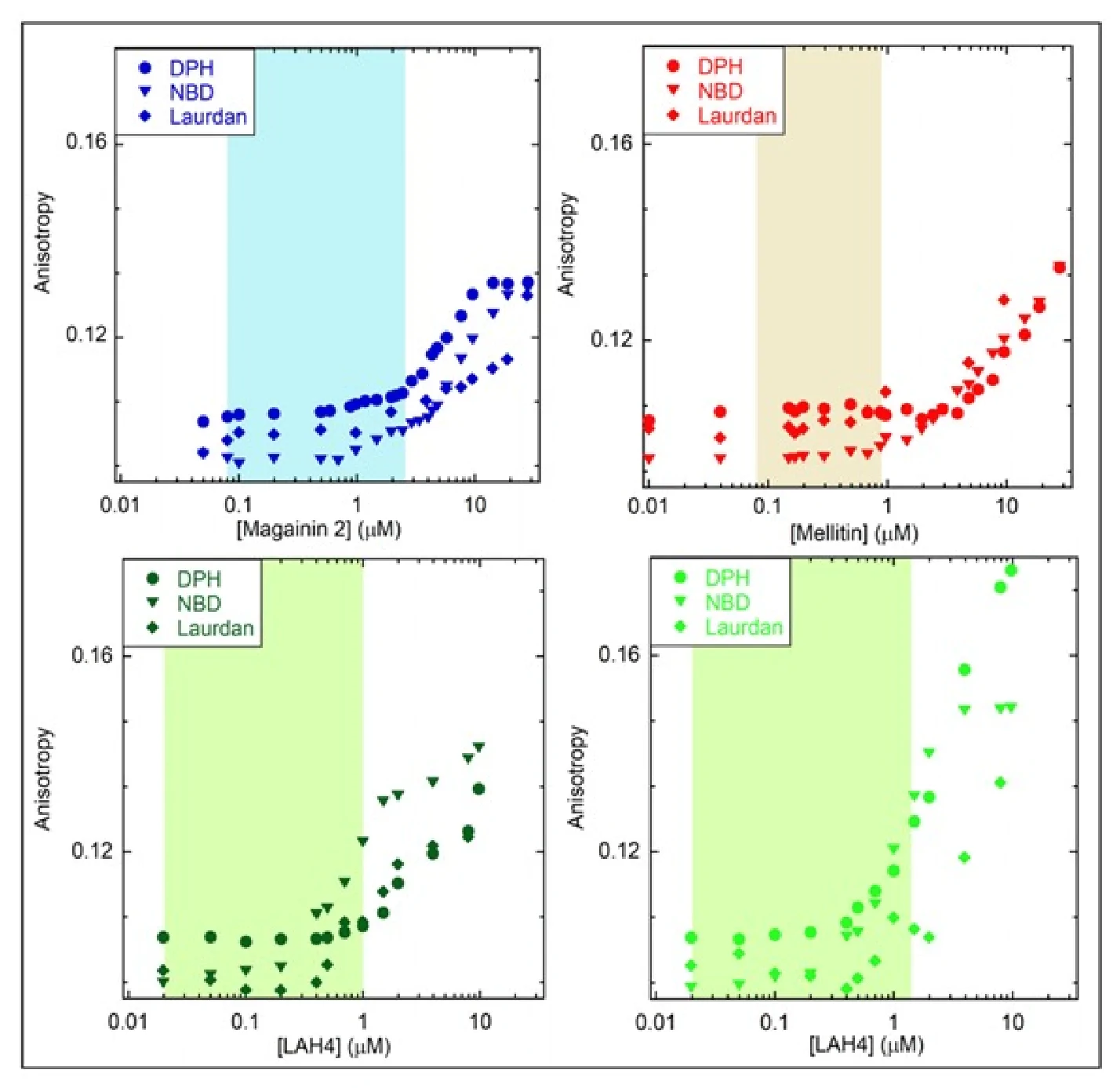
Steady-state anisotropy of membrane probes as a function of peptide concentration for magainin 2 (**upper left panel**), melittin (**upper right panel**) and LAH4 (pH 5.0 (**bottom left panel**); pH 7.4 (**bottom right panel**)). Lipid vesicles composition: POPC/POPG (2:1 molar ratio, 1% probe), lipid concentration 50 µM. DPH: λ_exc_ = 372 nm, λ_em_ = 450 nm; NBD: λ_exc_ = 460 nm, λ_em_ = 530 nm, Laurdan: λ_exc_ = 372 nm, λ_em_ = 475 nm. Bandwidth = 2 nm and 4 nm for excitation and emission slits, respectively. The peptide concentration range that induced membrane leakage from >3% to >97% is reported as a shaded area, for comparison.

We employed three different fluorescent probes that are located at different depths into the lipid bilayer: DPH, NBD-PE, and Laurdan [40,41,42]. NBD-PE is the most superficial because the fluorophore is covalently linked to the phospholipid head group of POPE, and it is located at a distance of about 19–20 Ǻ from the bilayer center [40,43,44]. Particularly, depth-depending quenching (DDQ) data indicate that the probe explores a broad distribution of positions, with a maximum at around 15 Å from the bilayer center. Molecular dynamics (MD) simulations support this observation [45], suggesting a possible downward bending of the phosphoethanolamine group, with NBD inserting slightly below the headgroups [46]. For NBD, it is well known that homo-FRET can occur when two molecules are within the Förster radius [47,48], leading to a decrease in the anisotropy; the data in Figure 3 clearly show that the opposite effect is observed in our case. To further rule out this possibility, we repeated the anisotropy experiments with the membrane labeling reduced to 0.1% NBD-PE for the AMP melittin (from the 1% reported in Figure 3). The results were fully comparable, allowing us to exclude any artifacts related to homo-FRET (Figure S4). Unlike NBD-PE, the hydrophobicity of DPH places the probe much deeper within the bilayer, aligned parallel to the phospholipid hydrocarbon chains. Several approaches have been employed to estimate its location: DDQ experiments reported an average depth of 7.8 Å from the center of a PC membrane [42], a result consistent with MD simulations [49,50,51], which nevertheless reveal a distribution of sites within the hydrophobic core. Neutron scattering experiments [52] further suggest that a fraction of DPH molecules may localize closer to the membrane surface, though this population was not quantified. Laurdan, in contrast, resides at an intermediate depth, just below the phospholipid headgroups. DDQ experiments place its average position at 10–11 Å from the bilayer center [53,54], in agreement with MD simulations, which likewise reveal a distribution of positions within the bilayer [55,56].

All peptides cause an increase in the probe fluorescence anisotropy (Figure 3), slightly higher for LAH4 at neutral pH, probably for the peculiar trans-membrane configuration assumed by this peptide. Overall, these trends indicate a decrease in fluorophore mobility during the excited-state lifetime. The anisotropy trend is similar across the three probes, regardless of their position within the membrane, suggesting that the peptide exerted a global effect on the bilayer.

The reduction in probe mobility begins in the same concentration range as the peptide’s permeabilization activity, but the main effect occurs after the AMP activity is complete.

To ensure liposomal integrity is maintained even at this high peptide/lipid ratio, we performed dynamic light scattering (DLS) measurements on vesicle suspension before and after the addition of the maximum concentration of membrane-active molecules. The DLS experiments were carried out on POPC/POPG 2:1 molar ratio vesicles, conditions that for Killer-FLIP slightly differ from those used elsewhere. The results confirm that the compounds do not cause vesicle disruption (Figure S6). Furthermore, these data indicate that liposome aggregation is either absent or involves only a small fraction of the liposomes for all the compounds analyzed at the tested concentrations. For LAH4 at neutral pH and Killer-FLIP, a slight increase in the average liposome size is observed, likely due to liposome fusion. Accordingly, caution should be exercised when interpreting the results presented in this study for these two compounds.

### 2.3. Perturbation of Membrane Fluidity and Order: Time-Resolved Anisotropy

Increased steady-state anisotropy can be caused by a reduction in the amplitude and/or rate of the probe motions, by a shortening of the fluorescence lifetime or by both variations [57]. To discriminate among these contributions, we performed time-resolved fluorescence intensity and anisotropy experiments both with and without peptides, using an AMP concentration that induced leakage close to 100%.

**Figure 4:**
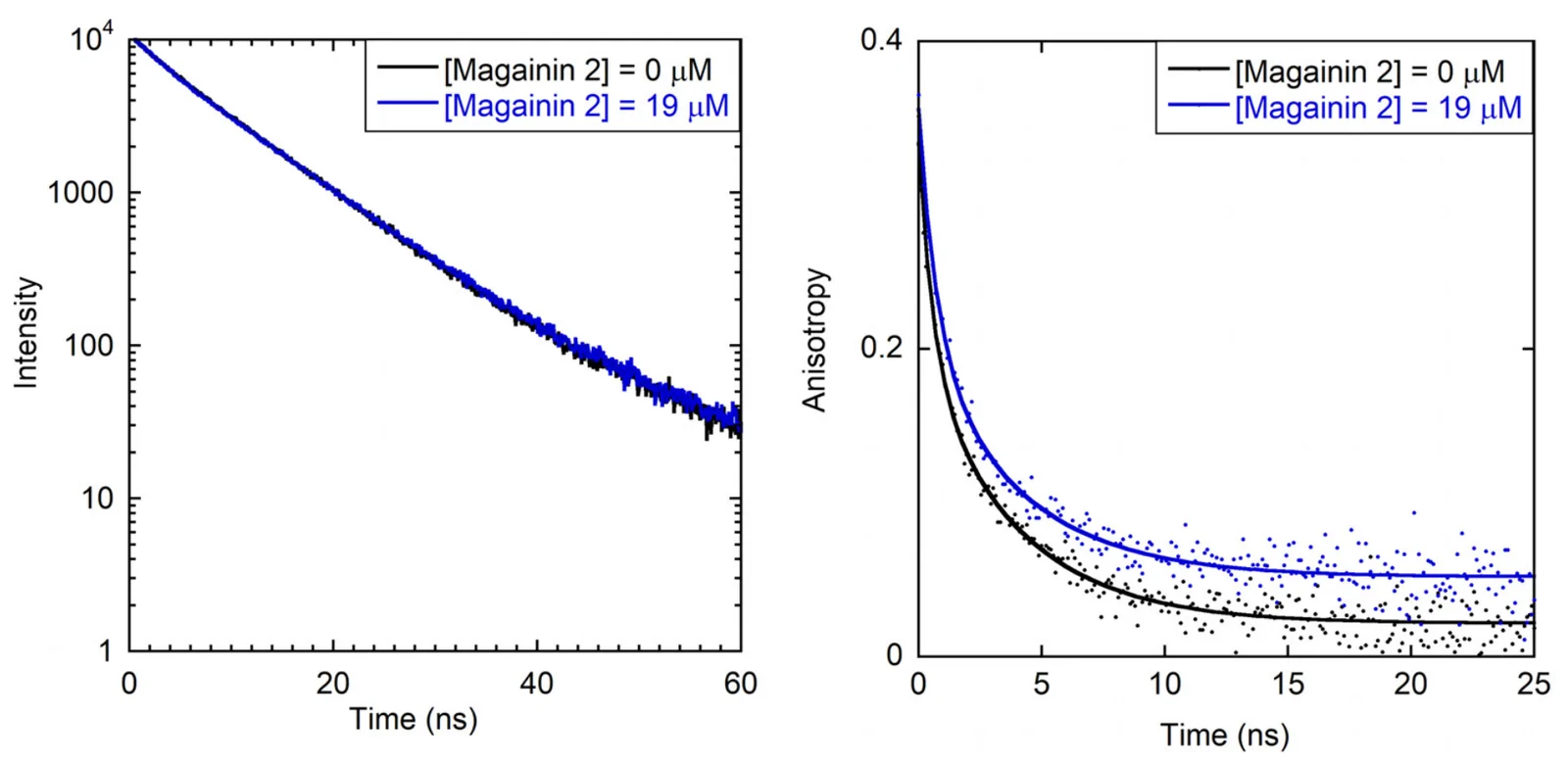
Fluorescence intensity decay (**left panel**) and time-resolved anisotropy (**right panel**) of NBD-PE inserted into POPC/POPG (2:1 molar ratio) vesicles (lipid concentration = 50 μM), without peptide (black points) and with magainin 2 19 μM (blue points). Laser λ_exc_ = 440 nm and λ_em_ = 530 nm with filter 495 nm. Anisotropy data were fitted with biexponential decay (continuous lines).

Representative data for fluorescence intensity decay and time-resolved anisotropy, respectively, are reported for liposomes labelled with NBD-PE in the presence of magainin 2 (Figure 4) at the highest concentration tested in steady state anisotropy measurements; similar results were obtained for all the other peptides and dyes. Peptide addition did not cause any variation in the fluorescence intensity decays, while the anisotropy decays were significantly affected.

Time-resolved anisotropy data were satisfactorily fitted by a biexponential decay using Equation (2), which describes hindered rotation, such as that of a probe embedded within a membrane bilayer. Here, $r_{0}$ is the limiting anisotropy (i.e., the anisotropy of the probe in the absence of rotational diffusion), $\phi_{i}$ represents the individual rotational correlation times, $f_{i}$ is the fractional amplitude of each correlation time, and $f_{\infty }$ denotes the residual fractional amplitude. These parameters allow the calculation of both the average rotational correlation time $\left\langle \phi \right\rangle$ (Equation (3)) and the absolute residual anisotropy $r_{\infty }$ (simply obtained by multiplying $r_{0}\times f_{\infty }$), which indicate how fast the probe rotates and how hindered its motion is, respectively.

**Figure 5:**
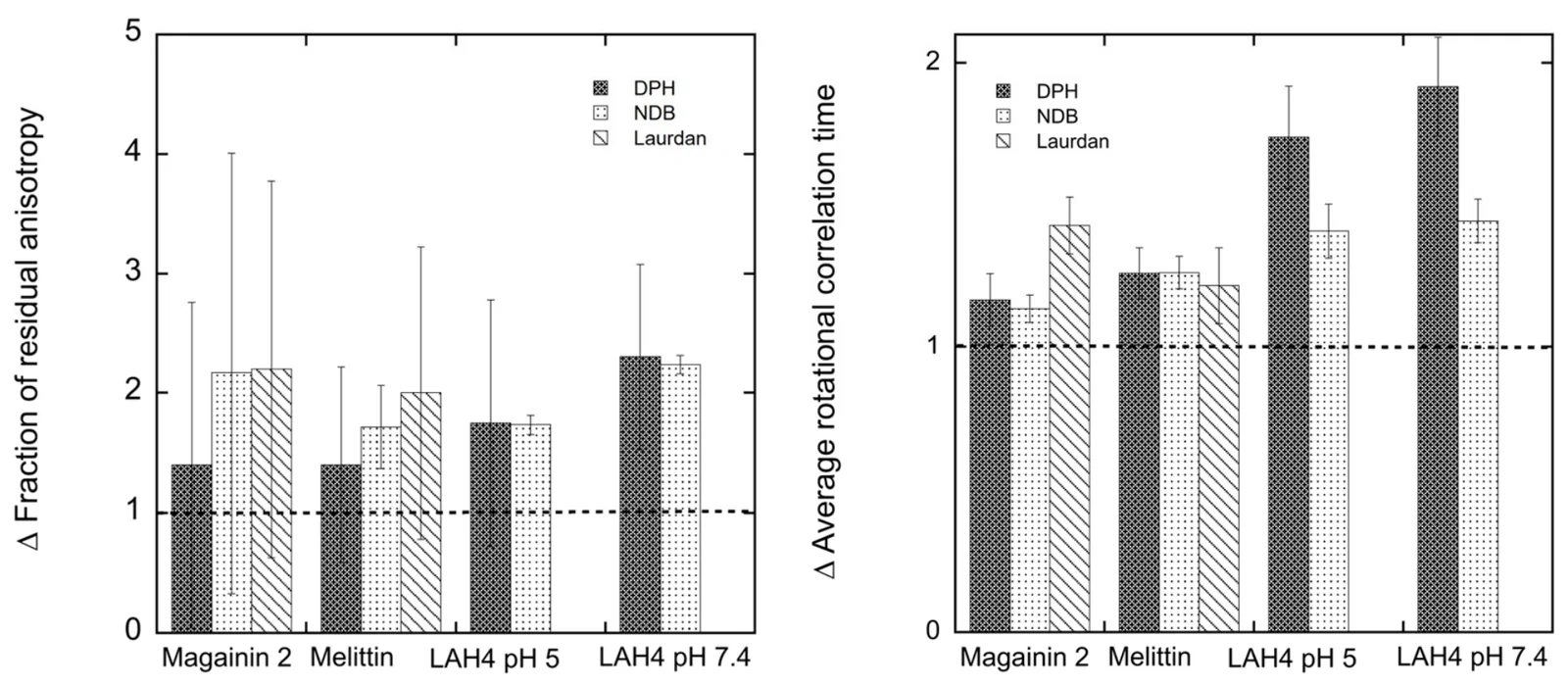
Relative increase of fraction of residual anisotropy (**left**) and rotational correlation time (**right**) of DPH, NBD, and Laurdan after peptides’ addition.

Data analysis of the biexponential fit for all the probes and peptides is reported in Supplementary Table S1. These measurements validate the results obtained with steady-state anisotropy: Although the errors are relatively high, the addition of all the tested peptides undoubtedly causes an increase in both the fraction of residual anisotropy and the average rotational correlation time (*f*_∞_) and <*ϕ*> in Equations (2) and (3), respectively (Figure 5; the absolute values are reported in Table S1).

This behavior indicates that the probe motions are both slower and more hindered in the presence of the peptide.

### 2.4. Water Penetration

**Figure 6:**
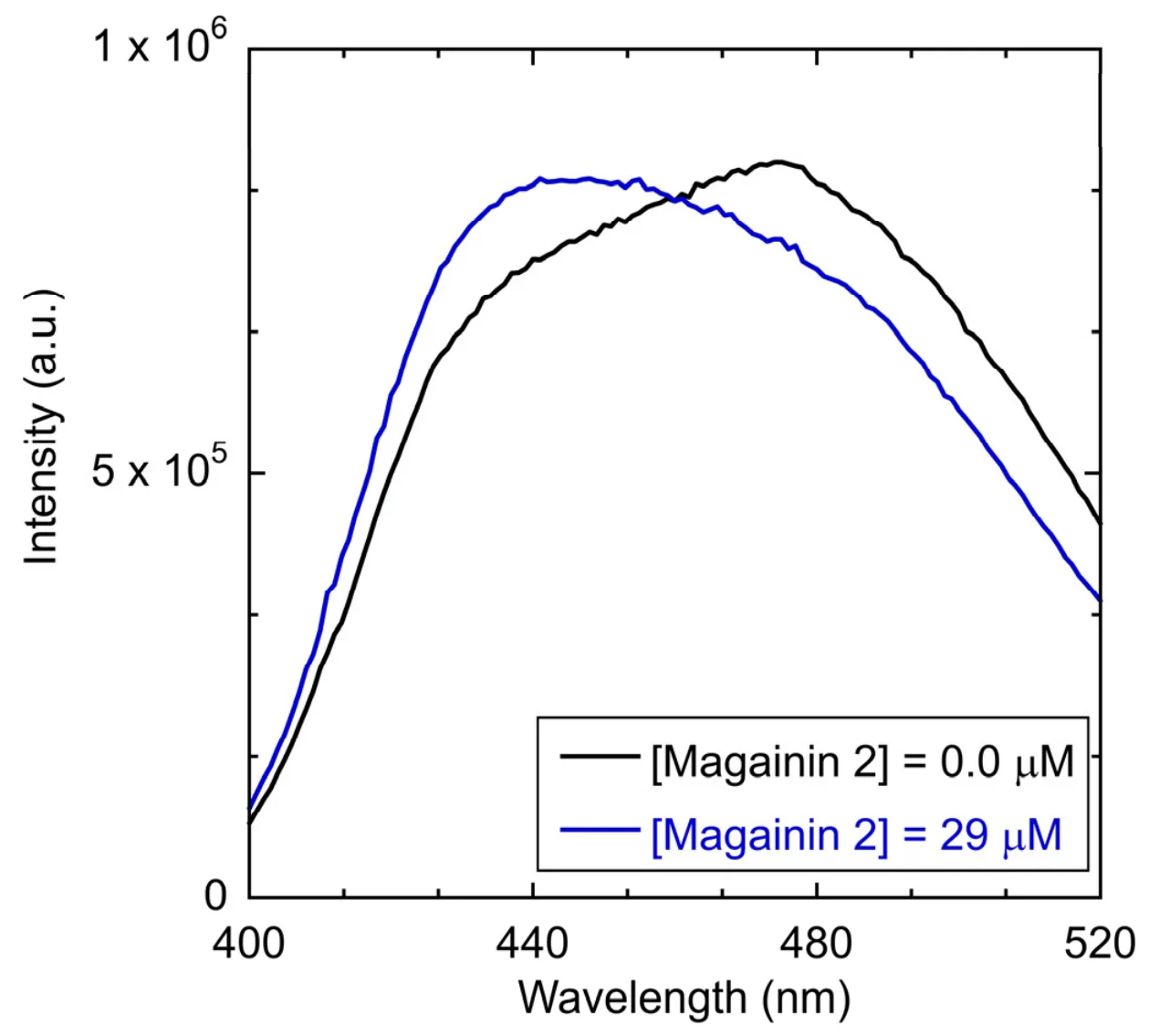
Changes in the fluorescence spectra of Laurdan in POPC/POPG vesicles (lipid concentration = 50 μM, 2:1 phospholipids molar ratio, probe 1%) upon addition of peptide. λ_exc_ = 372 nm, bandwidth = 3 nm, and λ_em_ = 475 nm, bandwidth = 4 nm.

Polar solutes such as water molecules can diffuse through the polar headgroups of the phospholipid membrane. Permeation through the bilayer is particularly favored at higher temperatures, when the lipid membrane undergoes a phase transition from the liquid-disordered to the liquid-ordered phase. Laurdan fluorescence is highly sensitive to solvent relaxation. When the water molecules surround the naphthalene moiety, the maximum wavelength of the fluorescence spectrum moves from about 435 nm to about 475 nm (Figure 6 for magainin 2 and Figure S8 for melittin) [58]. These spectral variations can be quantified by calculating the generalized polarization (GP) parameter, which has positive values when the spectrum is blue-shifted (apolar environment), and negative values when it is red-shifted (polar environment). GP is defined by analogy to fluorescence polarization, according to Equation (4) in Section 4 [59].

**Figure 7:**
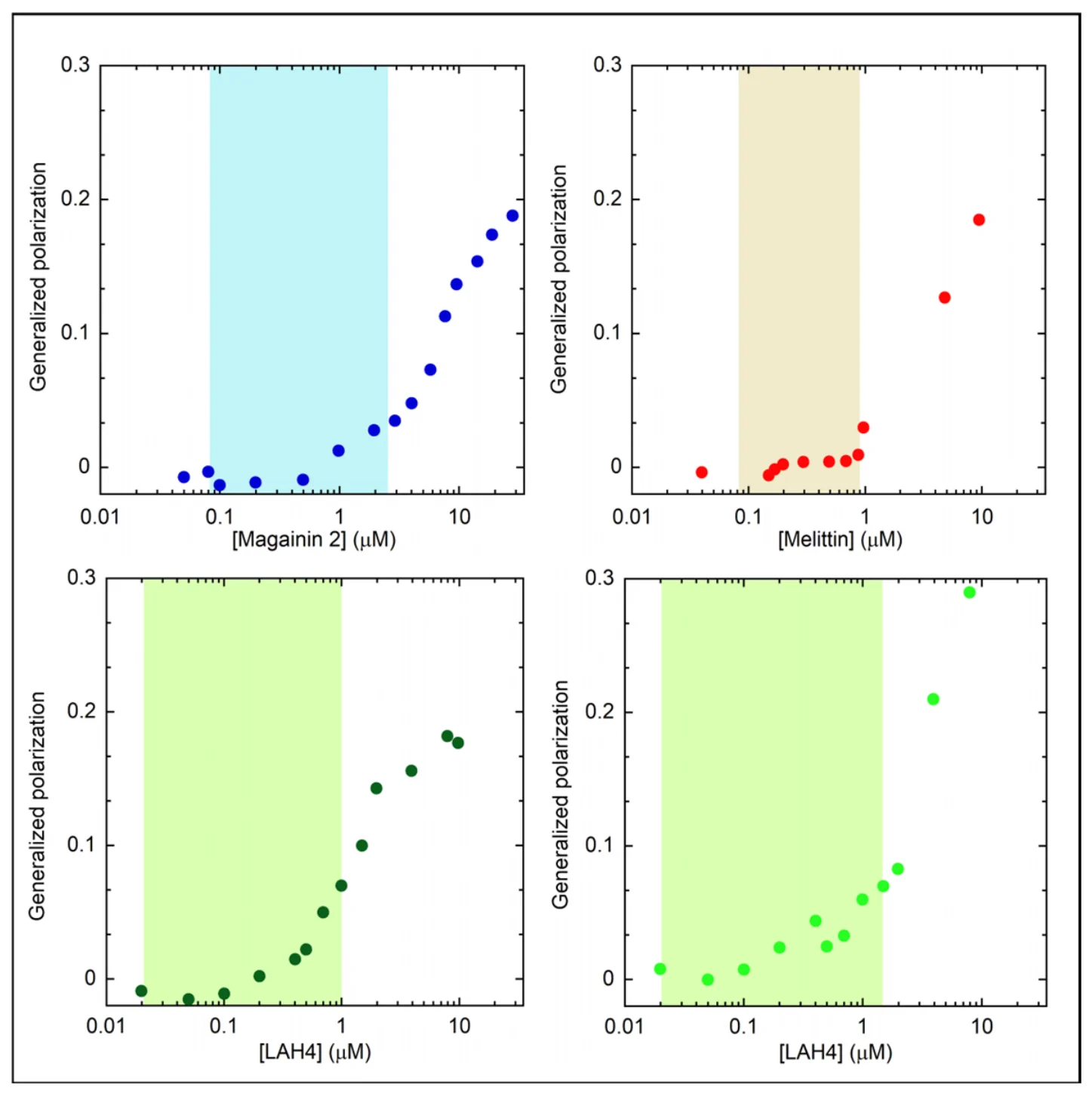
Generalized polarization of Laurdan fluorescence emission resulting from vesicles’ titration with magainin 2 ((**upper left panel**), lipid concentration = 50 μM, vesicle composition POPC/POPG 2:1 molar ratio, probe 1%), melittin (**upper right panel**) and LAH4 (**bottom left panel**) pH = 5.0 and (**bottom right panel**) pH = 7.4. λ_exc_ = 372 nm, bandwidth = 2 nm, λ_em_ = 400–580 nm, bandwidth = 1.5 nm, cut-off filter 385 nm. The peptide concentration range that induced membrane leakage from >3% to >97% is reported as a shaded area, for comparison.

Considering GP as a function of peptide concentration, both magainin 2 and melittin caused a reduction in water penetration into the lipid head group region (Figure 7). This finding is consistent with the membrane stiffening observed in the anisotropy measurements. The same behavior was observed for LAH4 at both pH values.

### 2.5. Lipid Lateral Mobility

Lateral diffusion is another key dynamic feature of the bilayer, as it is indirectly related to lipid-domain formation and stability and also affects the bidimensional diffusion of molecules in the bilayer plane. To investigate how the peptides affect this property, we exploited photophysical properties of pyrene. This fluorophore can form excited-state dimers (or excimers) when an excited and a ground-state molecule come in contact. Excimers are fluorescent, but their emission spectra differ from that of the monomer and shift to longer wavelengths. Excimer formation depends on the local concentration of pyrene molecules and on their mobility, since it is a diffusion-limited process. Therefore, the fluorescence of lipids labelled with pyrene can give information on the lipid diffusion; in particular, we measured the ratio between the intensities of the excimer and monomer emission bands, peaked at 475 nm and 397 nm, respectively (E/M ratio). A decrease in the lateral mobility of lipids will be reflected in a lower value of the ratio between the emission bands of excimer and monomer [60,61].

**Figure 8:**
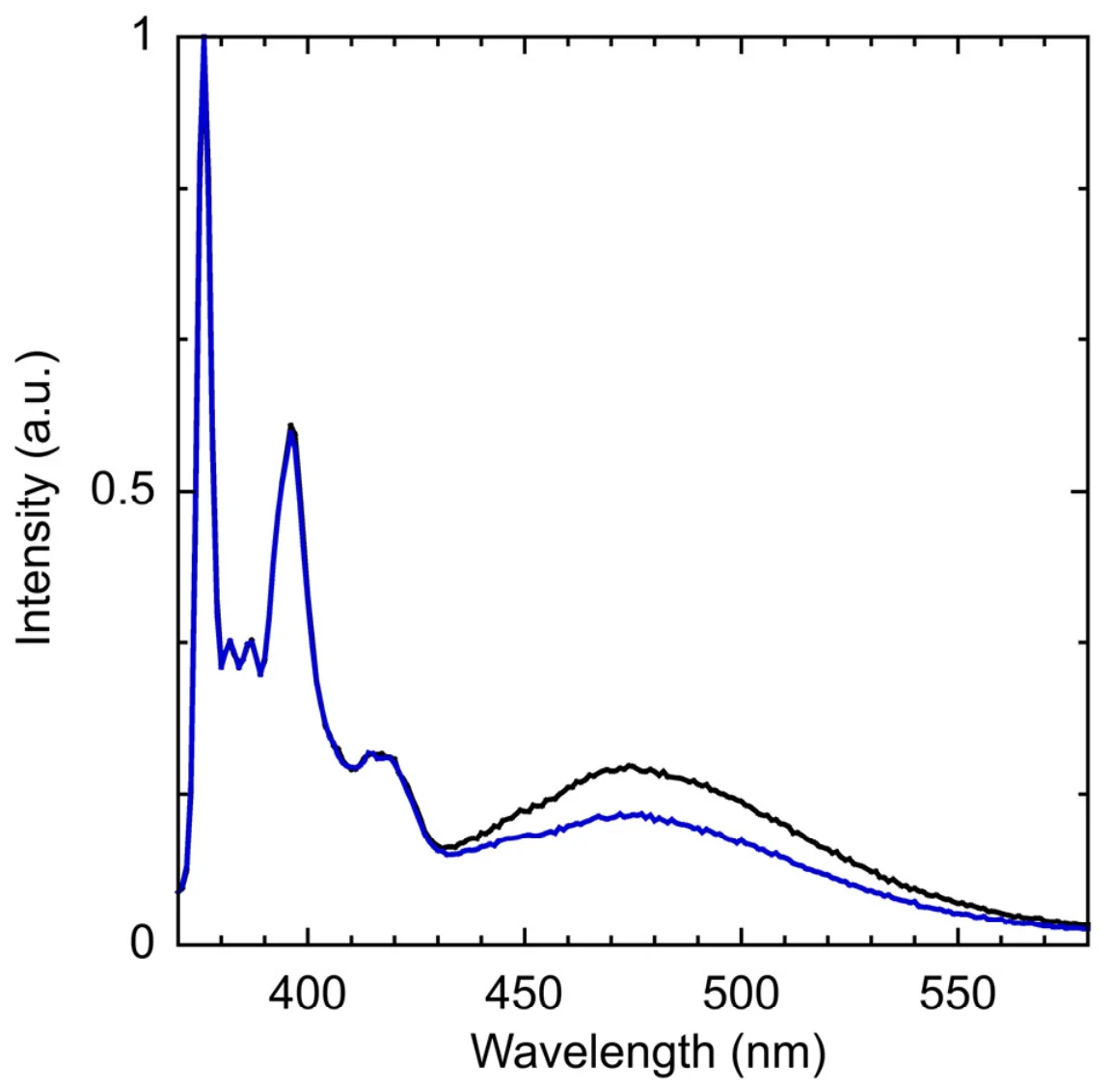
Normalized pyrene spectra in the absence (black line) and after addition of peptide (magainin 2: 29 µM, blue line) to POPC/POPG/Pyr-PC 2:1 molar ratio vesicles (lipid concentration = 50 µM, probe 3%). Pyrene: λ_exc_ = 328 nm, bandwidth = 1 nm, λ_em_ = 370–600 nm, bandwidth = 1.5 nm.

**Figure 9:**
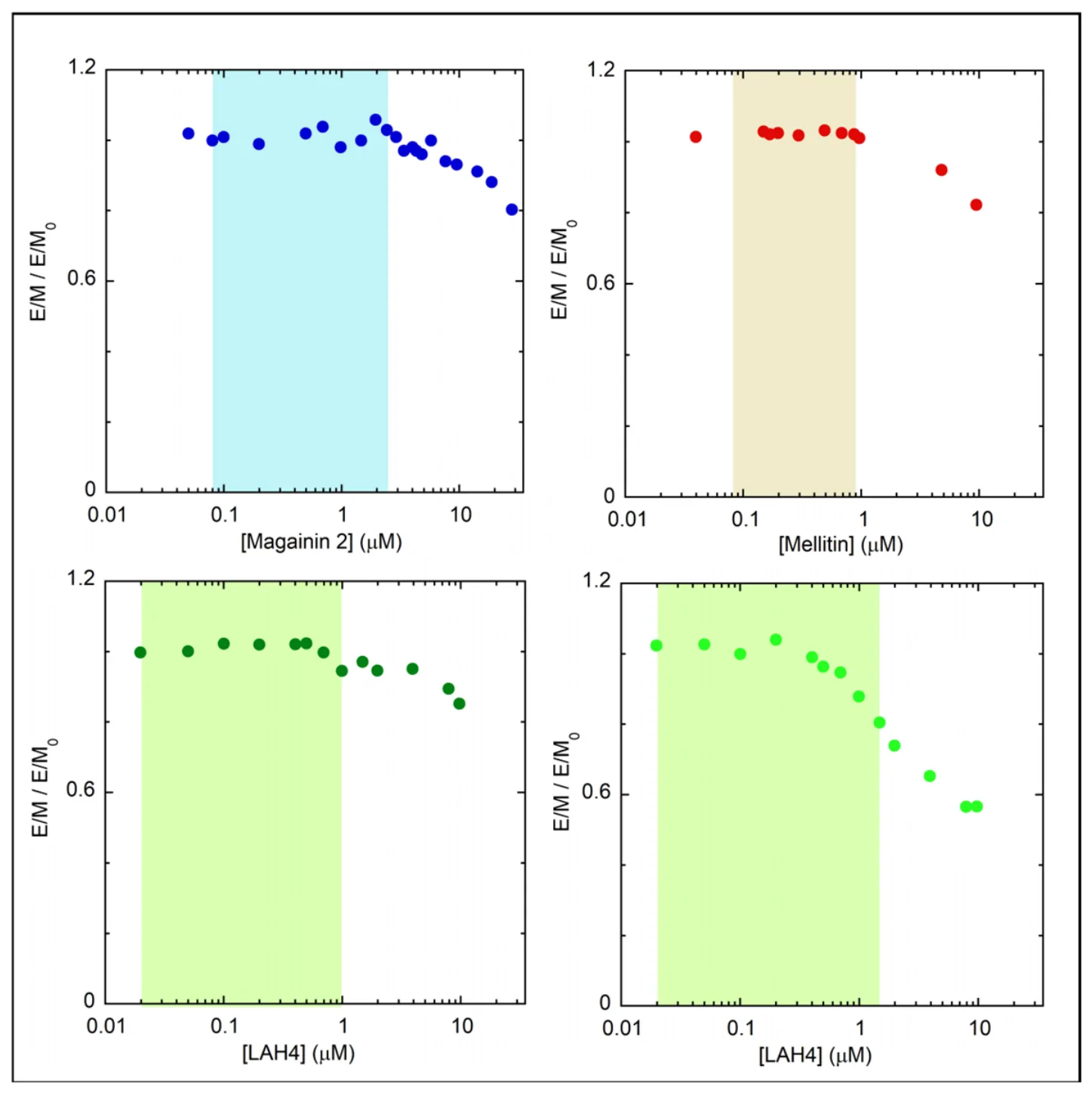
Variations in the ratio of the fluorescence emission intensities of the excimer (475 nm) and monomer (397 nm) bands caused by titration of POPC/POPG/Pyr-PC 2:1 molar ratio vesicles (lipid concentration = 50 µM, probe 3%) with magainin 2 (**top left panel**), melittin (**top right panel**), and LAH4 at pH = 5.0 (**bottom left panel**) and pH = 7.4 (**bottom right panel**). The peptide concentration range that induced membrane leakage from >3% to >97% is reported as a shaded area, for comparison.

Pyrene fluorescence spectra were recorded by titrating a liposome suspension (containing 3% of PC chain covalently labeled with pyrene) with increasing peptide concentrations. As an example, the relative intensity of excimer emission of pyrenyl-lipid decreased after the addition of magainin 2 is reported, indicating a decrease in the lateral mobility of the phospholipids (Figure 8). E/M ratios at different peptide concentrations are reported in Figure 9 for all the peptides investigated. The data demonstrated a decrease in excimer emission and thus a lower lipid lateral mobility after peptide addition. This effect was most evident at peptide concentrations above those required to cause leakage.

**Figure 10:**
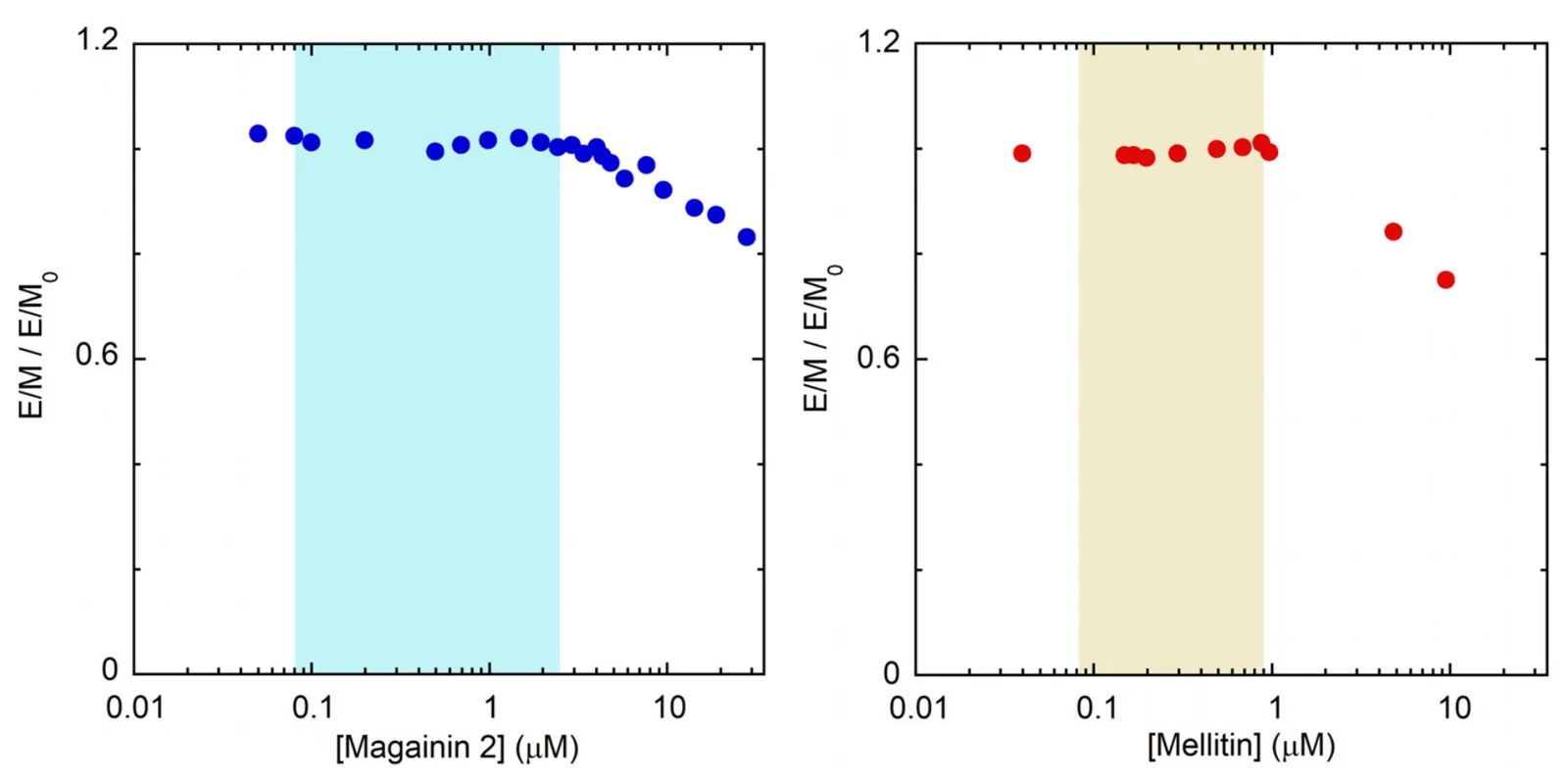
Variations in the ratio of the fluorescence emission intensities of the excimer (475 nm) and monomer (397 nm) bands caused by titration POPC/POPG/Pyr-PG (66:30:3) vesicles (lipid concentration = 50 μM) with magainin 2 and melittin. The peptide concentration range that induced membrane leakage from >3% to >97% is reported as a shaded area, for comparison.

Lipid vesicles employed so far were labelled with pyrene covalently linked to the PC hydrocarbon chain. To rule out the possibility that the observed effect was due to a specific interaction of the peptide with the zwitterionic lipid, the same experiment was performed with magainin 2 and melittin using the pyrene probe linked to the anionic lipid phosphatidylglycerol (POPG). A very similar trend was observed, indicating that the reduced mobility of the probe is not due to a direct interaction between peptide and the labelled lipid (Figure 10) but is a general AMP-induced membrane effect.

### 2.6. Vesicle Agglutination

Peptide-induced vesicle agglutination is a common observation with cationic AMPs [13]. Therefore, we studied this process for representative compounds used in the manuscript to verify whether it played any role in the perturbation of membrane dynamics observed in the experiments reported in the previous subsections. Here, we show data for magainin 2. Data for melittin, Nor-Leu, and Nor-Phe, confirming the discussed picture, are reported in Figure S7. Aggregation was followed by measuring the increase in absorbance at 600 nm due to light scattering caused by sample turbidity.

**Figure 11:**
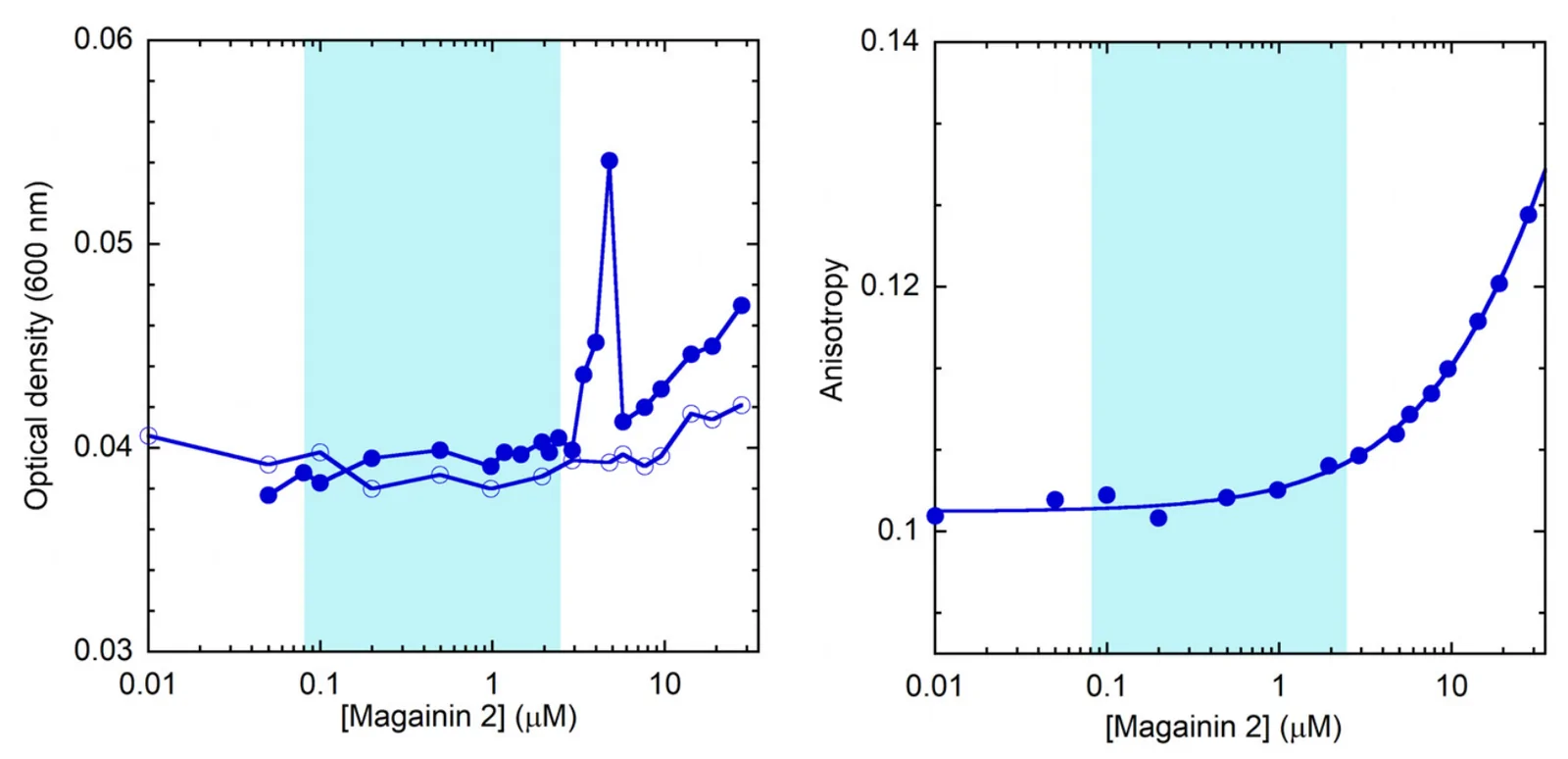
(**Left**): peptide-induced POPC/POPG (2:1 molar ratio) liposome agglutination as measured from turbidity. Optical density at 600 nm of vesicles (lipid concentration = 50 μΜ) in the presence of PEG-PE (empty circles) and without it (full circles), titrated with increasing magainin 2 concentrations. (**Right**): DPH anisotropy inserted into liposomes with PEG-PE (POPC, POPG, PEG-PE, DPH; 66, 31, 2, 1 mole %, respectively) resulting from titration with varying concentrations of magainin 2. Lines serve only as a guide for the eye. The peptide concentration range that induced membrane leakage from >3% to >97% is reported as a shaded area, for comparison.

Like in the other experiments, a suspension of POPC/POPG liposomes (2:1 molar ratio, lipid concentration = 50 µM) was titrated with increasing magainin 2 concentrations (Figure 11). At approximately 5 μM of peptide, a sharp increase in apparent absorbance was observed, followed by a rapid reversal of this phenomenon. This behavior is consistent with what has been previously observed for similar systems [13]. When the peptide binds to the anionic membranes, its positive charges progressively neutralize the negative charges of the lipids, favoring the formation of aggregates. When more peptide molecules are added, the process is reversed, since the total net charge becomes positive, causing repulsion between vesicles and a reversal in the aggregation.

It is thus reasonable to question if all the variations in membrane dynamics reported in the previous sections arise from the peptide-induced perturbation of the bilayer or are simply a consequence of vesicle agglutination. To clarify this point, we used steric repulsion between vesicles to reduce their aggregation, by including in the lipid mixture phospholipids derivatized at their head group with the hydrophilic polymer polyethylene glycol (PEG) (POPC, POPG, PEG-PE, DPH; 66, 36, 2, 1 mole %, respectively). This approach essentially eliminated liposome agglutination (Figure 11, left panel). By contrast, the increase in DPH anisotropy was comparable to that previously observed in POPC/POPG vesicles (Figure 11, right panel), ruling out the possibility that the peptide-induced membrane stiffening is an artifact arising from vesicle agglutination.

### 2.7. Bioactive Compounds: Influence on Membrane Dynamics

There is an increasing trend toward shifting from antimicrobial peptides to the design of peptidomimetics that perfectly mimic the behavior of AMPs toward biological membranes. The advantage of these compounds lies in their greater stability and lower synthesis costs compared to peptides. Growing evidence indicates that these peptidomimetics behave similarly to AMPs in their ability to disrupt membranes [28]. It is worth asking whether they are also capable of employing additional or alternative mechanisms that could lead to greater membrane permeabilization efficacy by inducing a global perturbation of the bilayer dynamics, as observed in this work for AMPs. To assess whether our findings apply more broadly to structurally different membrane-active compounds and not only to AMPs, we extended the same spectroscopic studies to other membrane-active molecular classes, including the anticancer peptide Killer-FLIP [30] and the two norspermidine-based peptidomimetics, Nor-Leu and Nor-Phe [28]. Like AMPs, they disrupt the phospholipid bilayer by creating defects, compromising its integrity and leading to membrane perturbation and ultimately cell death (see Figures S2 and S3 for the membrane-perturbing activity of these compounds).

**Figure 12:**
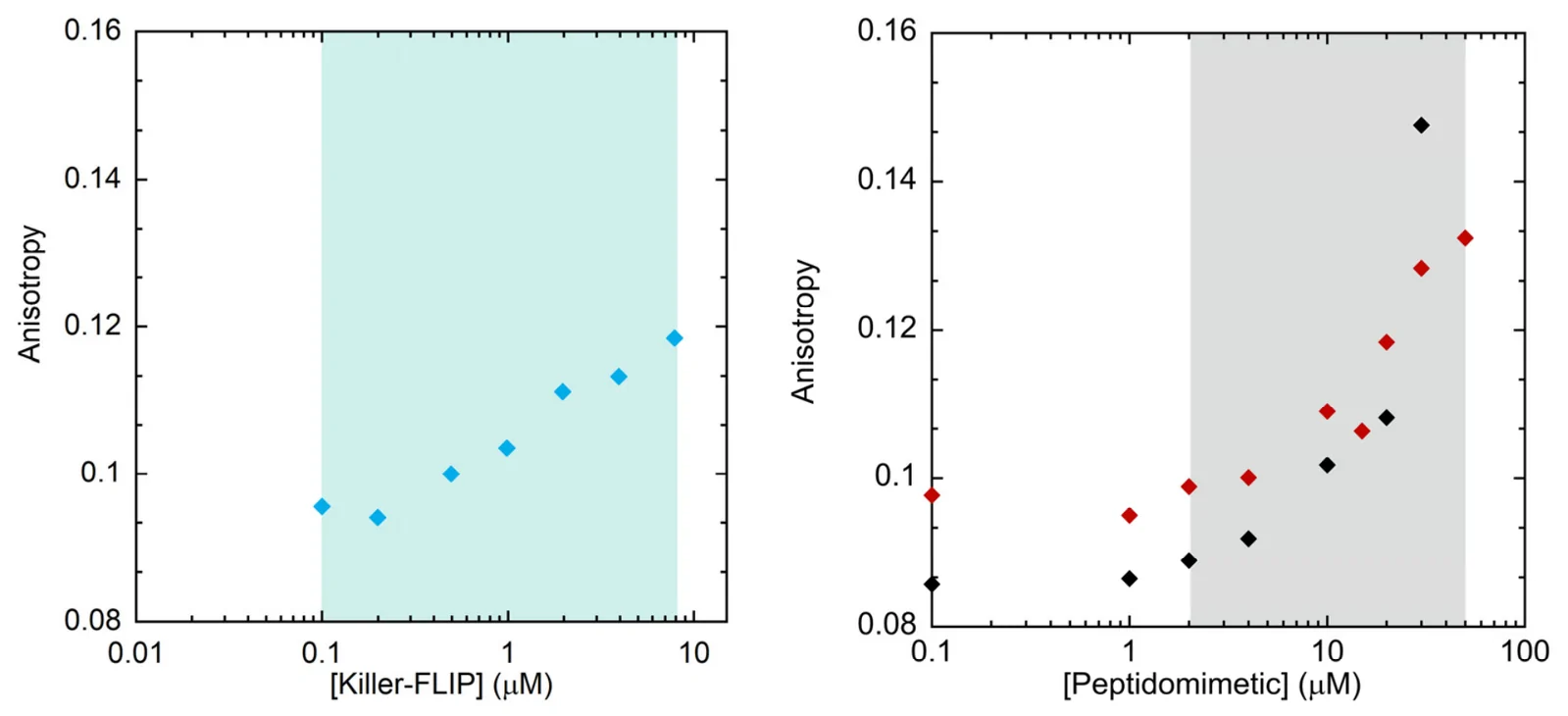
Steady-state anisotropy of Laurdan resulting from vesicles titration with killer-FLIP ((**left panel**), lipid concentration = 50 μM vesicle composition POPC/POPS 1:1 molar ratio—better mimicking the membranes in cancer cells) and peptidomimetics Nor-Leu and Nor-Phe ((**right panel**), black: Nor-Phe, red: Nor-Leu. Vesicle composition POPC/POPG 2:1 molar ratio). Laurdan λ_exc_ = 372 nm, bandwidth = 2 nm, λ_em_ = 475 nm, bandwidth = 1.5 nm, cut-off filter 385 nm. The compound concentration range that induced membrane leakage from >3% to >97% is reported as a shaded area, for comparison.

**Figure 13:**
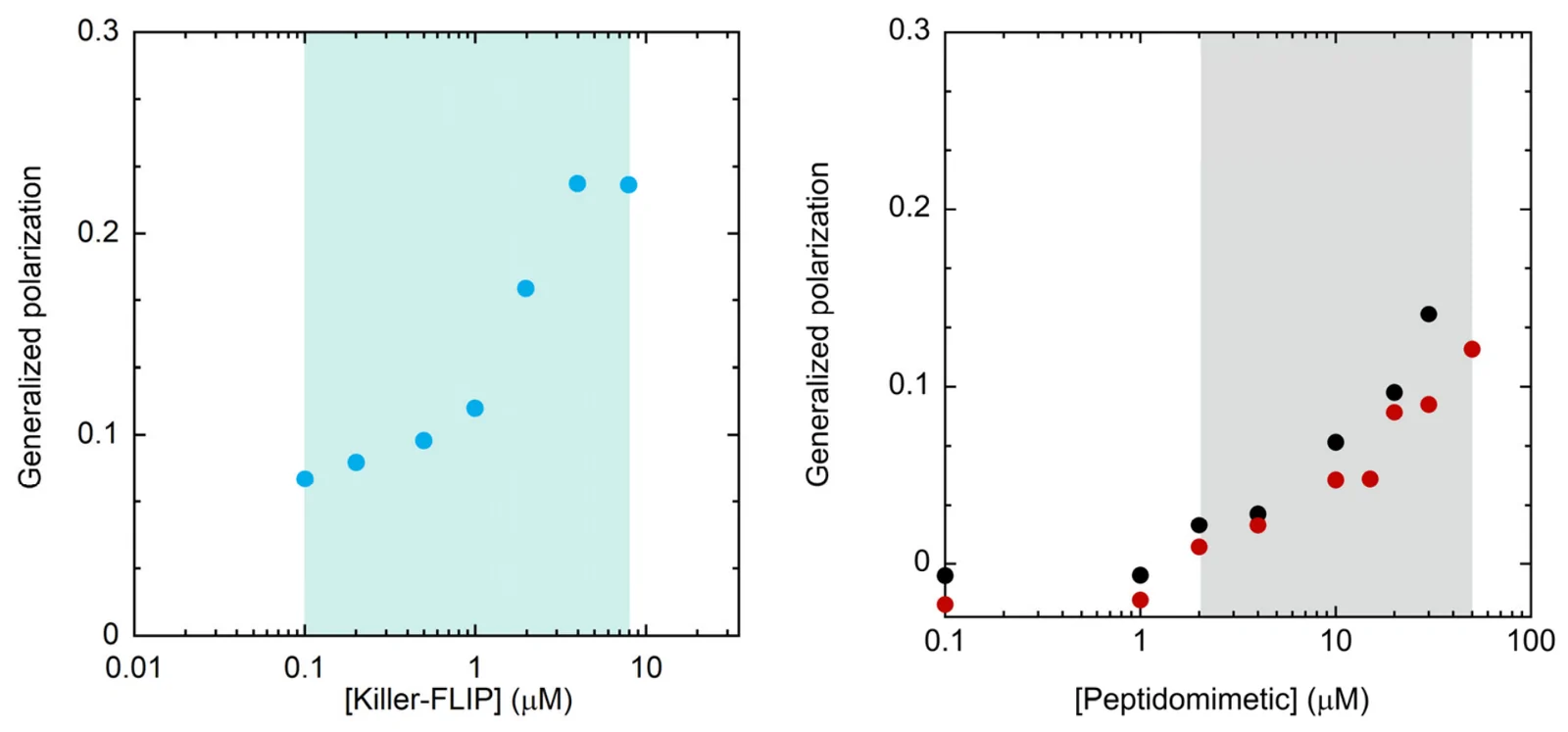
Generalized polarization of Laurdan resulting from vesicles’ titration with killer-FLIP ((**left panel**), lipid concentration = 50 μM, vesicle composition POPC/POPS 1:1 molar ratio) and with peptidomimetics Nor-Leu and Nor-Phe ((**right panel**), black: Nor-Phe, red: Nor-Leu. Vesicle composition POPC/POPG 2:1 molar ratio). Laurdan λ_exc_ = 372 nm, bandwidth = 2 nm, λ_em_ = 475 nm, bandwidth =1.5 nm, cut-off filter 385 nm. The compound concentration range that induced membrane leakage from >3% to >97% is reported as a shaded area, for comparison.

As previously reported, we focused on steady state anisotropy (Figure 12) and generalized polarization measurements of the Laurdan probe (Figure 13).

Killer-FLIP is mainly studied for its anticancer properties. A different membrane composition (POPC/POPS 1:1) was used to better mimic the membrane composition of cancer cells, in which phosphatidylserine (PS) is no longer confined to the inner leaflet of the membrane but becomes exposed to the outer surface. This leads to a more negative charge than in healthy cells, making their membrane more similar to those in bacteria [62,63,64,65] in this regard.

Also for these classes of molecules, an increase in anisotropy is observed, indicating that the probe is surrounded by a more viscous environment after the bioactive compound addition. Of note, in these cases the effect on the membrane fluidity takes place in the same range of permeabilization, leaving open the question of whether the bactericidal properties are due to a concomitant mechanism in which both the membrane permeabilization and effect on the lipid dynamics play a role.

The generalized polarization of the probe is affected as well. The interaction of Killer-FLIP, Nor-Phe and Nor-Leu with model membranes causes a decrease in the hydration of the bilayer, as shown by the increase in GP values.

### 2.8. Comparison Between Membrane-Active Peptides and Peptidomimetics

Results obtained so far indicate that both membrane-active peptides and peptidomimetics induce stiffening of the entire bilayer in model membranes. However, to directly compare the effects of the two classes of compounds on membrane dynamics, further considerations are necessary. Peptides analyzed so far range from 23 residues for magainin 2, to 26 residues for melittin and LAH4, and 18 residues for killer-FLIP. Consequently, a normalization for the different surface of each molecule can help to account for effects due to mere peptide higher dimension.

From molecular dynamics simulations, it is known that the well-studied antimicrobial peptide PMAP-23, which is 23 residues long, occupies a surface area of 5.4 nm^2^ when folded into its helical conformation [11]. The AMPs analyzed in this work are comparable in terms of length, net charge, and secondary structure; therefore, the same surface area of 5.4 nm^2^ was assumed for the peptides investigated here, but the value was further normalized to take into account the slight difference in length between PMAP-23 and the peptides investigated in this study (see Section 4.10).

Similarly, for the nor-spermidine-based molecules, a surface area of 0.8 nm^2^ was considered. This value was calculated for a related nor-spermidine compound containing a tryptophan residue, instead of phenylalanine or leucine [28,66].

**Figure 14:**
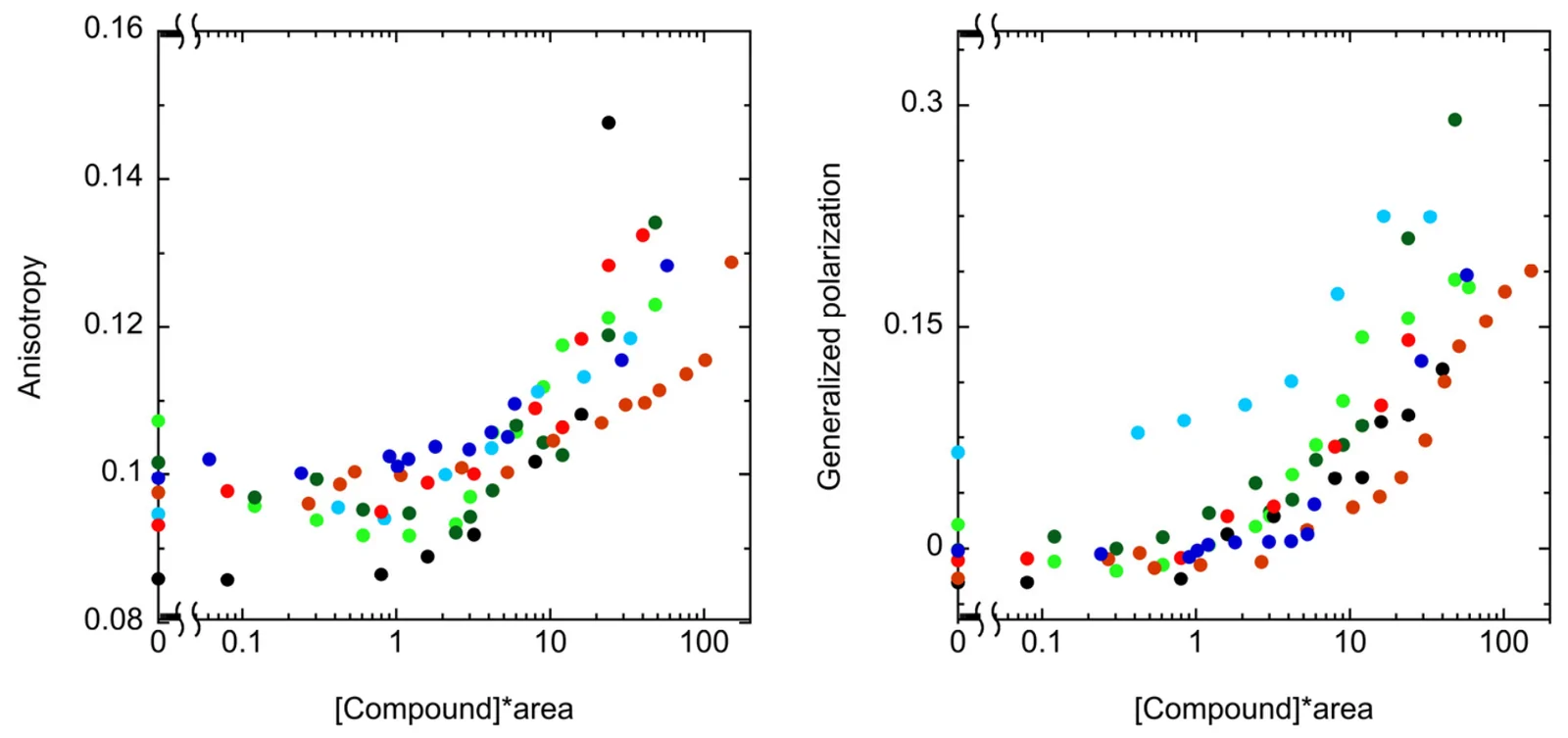
Comparison of steady-state anisotropy (**left panel**) and GP (**right panel**) of Laurdan after the addition of all the AMPs and peptidomimetics used in the present work (melittin: blue; magainin 2: orange; LAH4 pH 5: green; LAH4 pH 7.4: light green; Killer-FLIP: light blues; Nor-Leu: red; Nor-Phe: black). Concentrations were normalized for the surface area occupied by AMPs or peptidomimetics. Vesicles’ composition: POPC/POPG at a 2:1 molar ratio for all compounds, except for Killer-FLIP, for which a POPC/POPS 1:1 molar ratio was employed. The symbol * indicates a multiplication of the compound concentration by the occupied area.

When the concentrations of peptides and peptidomimetics were rescaled to account for the surface area of each molecule, the effects on membrane dynamics occurred within the same range for all the compounds tested, indicating a general behavior of membrane-active molecules (Figure 14), independently of possible different configurations of the analyzed peptides into the bilayer. The only compound showing a slight deviation from the others is Killer-FLIP, for which the GP parameter exhibits higher values. However, it should be noted that the membrane composition in this case differs from the others, which could account for the observed differences in terms of GP behavior, since both the Laurdan fluorescence features [67,68] and the response to the interaction with peptides [69] are strongly dependent on the membrane composition.

Table 1 and Table S2 (see Section 4.10 for details) report the molecular footprint for all the peptides, calculated as described above, for which values in the range 4.2–6.1 nm^2^ were obtained. Starting from these data and assuming an estimated average fluid-phase surface area of 0.63 nm^2^ per lipid for POPC/POPG and 0.59 nm^2^ per lipid for POPC/POPS (Table S2), the peptide concentrations required to completely cover both the external and internal vesicle surfaces can be estimated. For almost all the considered compounds, the concentrations at which effects on lipid dynamics are observed exceed these limit values, and complete liposome coverage can be predicted (Table S2). Slightly different conditions are observed for Killer-FLIP and the peptidomimetics Nor-Leu and Nor-Phe; in these cases, the concentrations used in this work are slightly lower than those required to fully cover both leaflets. Overall, these data indicate that, for the investigated compounds, to observe membrane stiffening, the liposome surface must be completely or almost completely saturated by the bioactive molecules.

## 3. Discussion

Bacteria exploit the diversity of their lipids to modify their membranes when facing environmental stress, thereby maintaining membranes in an optimal state and preserving their proper function. Several theories tried to explain this peculiar behavior. The homeoviscous adaptation was introduced by Sinensky, according to which the membrane undergoes a reconstruction to keep the bilayer fluidity at a constant value despite changing conditions (mainly temperature) [70]. In the same years, McElhaney proposed that it may not be necessary to maintain a precise level of fluidity; rather, organisms might adjust the temperature range of the thermal transition so that most lipids remain in the appropriate liquid-crystalline phase [71]. Later, Silvius [72] speculated that although cells cannot grow when their membranes are in the gel state, they can tolerate a broad spectrum of fatty acid composition and fluidity within the liquid-crystalline phase. Although the primary target of AMPs remains the bacterial membrane, increasing evidence indicates that they can act through multiple mechanisms to induce cell death [10,73,74].

Nowadays, it is widely accepted that membrane fluidity can change due to interactions between lipids and environmental factors such as temperature, pH, pressure, ions, water, nutrients, and chemicals. Alterations in one of these factors trigger bacterial adaptive responses, making membrane adjustments a key part of the multi-factorial stress adaptation process necessary for cell survival. In the present work, we investigated the behavior of membrane-active compounds with a particular focus on this aspect.

Our previous works demonstrated that antimicrobial peptide molecules must fully cover the bacterial cell membrane in order to exert their bactericidal activity [11,12]. At these very high peptide concentrations, the peptides interfere with the bilayer dynamics [13]. This behavior was observed in both model vesicles and live bacterial cells.

In the present study, we systematically extended these investigations on model membranes to membrane-active peptides and small molecules from different classes.

The peptidomimetics employed are cationic and amphiphilic. Despite their simple structures, these molecules have been shown to act similarly to antimicrobial peptides, primarily targeting the bacterial membrane. Their interaction with the bilayer is largely through binding to the membrane surface just below the head-group region, and insertion of their apolar moieties (amino acids side chains and fatty acid chains) into the bilayer core, leading to defect formation once a high level of membrane coverage is reached [33].

Anisotropy measurements conducted using three different probes, positioned at different depths and spanning the entire bilayer, indicate an overall increase in membrane viscosity.

Sporadic reports described effects on membrane dynamics similar to those discussed here, with different antimicrobial peptides. In particular, an increase in generalized polarization is observed, which typically correlates with reduced membrane fluidity in vesicles. Similar behaviors are observed when techniques on the nanosecond timescale, such as fluorescence and EPR spectroscopy, are used [75,76,77,78,79]; interestingly, an increase in fluidity is observed when NMR techniques (ms time scale) are used [29]. Further studies would be warranted to explain these differences. Here, we show that effects on bilayer fluidity occur at higher concentrations with respect to membrane leakage. The evidence that the GP values, likely due to probes spread on the whole liposome, remain unchanged at the concentrations in which leakage occurs, strongly suggests that membrane permeabilization involves only a small fraction of the bilayer. Very likely, a great part of the phospholipids remains far from the membrane-defect area, and this portion undergoes a reduction in fluidity. However, our results extend beyond membrane hydration, providing a broader view of the overall effects on membrane dynamics. The outcomes from viscosity measurements, membrane hydration, and lipid lateral mobility are in perfect agreement, all pointing to a peptide-induced membrane stiffening, at comparable or slightly higher concentrations than those needed to permeabilize the bilayer (1–10 µM). This suggests that membrane stiffening is not caused by the formation of defects within the bilayer, but rather by an almost complete coverage of the vesicle surface by the peptides [11,12]. Indeed, for several AMPs, very high peptide-to-lipid (P/L) molar ratios have been reported to induce membrane permeabilization, approaching complete membrane surface coverage in both liposomes and bacteria [80]. Based on the peptide footprint and the phospholipid surface area [11], the effects observed here occur at even higher peptide concentrations, consistent with the “carpet” mechanism previously described for the analyzed peptides.

Nor-Leu and Nor-Phe peptidomimetics appear to affect membrane dynamics at higher concentration ranges. However, considering the difference in molecular size between these compounds and AMPs, the effective activity range becomes narrower and closely aligns that of AMPs. Thus, even for the smaller peptidomimetics, once the full coverage of the vesicle surface is most likely reached, a crucial effect is the stiffening of the entire bilayer.

Lipid domain formation is sometimes recalled to explain the fluorescence signals indicating higher rigidity [68]. This alternative mechanism cannot be definitively ruled out; however, our data show that the same effect is obtained with three different probes placed at different depths into the bilayer. Excluding the unlikely possibility that all the probes are located in the more rigid domains, our evidence suggests a more general stiffening of the whole membrane.

Overall, the evidence that only minor effects on lipid dynamics are observed at concentrations leading to membrane permeabilization suggests that effect on the membrane dynamics, which parallel the so-called “sand-in-a-gearbox” mechanism for bacteria, does not play a significant role in liposome permeabilization for the system here investigated. Of note, in bacteria, changes in membrane fluidity could lead to a variation in lipid and protein packing, with possible implications on their overall physiological functioning. For this reason, the evidence here reported that leakage and influence on membrane fluidity are independent phenomena, taking place at different concentration ranges, strongly supports the idea to perform these kinds of measures in bacteria, too [13].

## 4. Materials and Methods

### 4.1. Materials

POPG (1-palmitoyl-2-oleoyl-sn-glycero-3-[phospho-rac-(1-glyc-erol)]), POPC (1-palmitoyl-2-oleoyl-sn-glycero-phosphocholine), POPS (1-palmitoyl-2-oleoyl-sn-glycero-3-phospho-L-serine), NBD-PE (L-α-phosphatidylethanolamine-N-(7-nitro-2-1,3-benzoxadiazol-4-yl)), and PEG-PE (1,2-distearoyl-sn-glycero-3-phosphoethanolamine-N-[methoxy(polyethylene glycol)-2000]) were purchased from Avanti Polar Lipids (Alabaster, AL, USA); Pyr-PC (1-palmitoyl-2-(pyrene-1-yl)dec-anoyl-sn-glycero-3-phosphocholine) and Pyr-PG (1-palmitoyl-2-(pyr-ene-y-yl)decanoyl-sn-glycero-3-phosphoglycerol) were obtained from Sigma Aldrich (St. Louis, MO, USA); DPH (diphenylhexatriene) and Laurdan (2-(dimethylamino)-6-dodecanoylnaphthalene) were purchased from Fluka (St. Louis, MO, USA). Spectroscopic-grade chloroform and methanol (Carlo Erba, Milano, Italy) were used. Triton X-100 was purchased from Acros (Geel, Belgium).

#### 4.1.1. Synthesis of Peptide and Peptidomimetics

The peptides LAH4, melittin and magainin 2 were prepared by solid-phase peptide synthesis on Millipore 9050 (Millipore Corp., Bedford, MA, USA) or ABI433 (Applied Biosys-tems, Weiterstadt, Germany) automatic peptide synthesizers using Fmoc chemistry. A fourfold excess of Fmoc-protected amino acids (Bachem, Heidelberg, Germany) was used during chain elongation. After TFA cleavage was achieved, peptides were purified by preparative HPLC using an acetonitrile/water gradient in the presence of 0.1% TFA and a 214 nm detection wavelength. The identity and high purity of the products were confirmed by MALDI mass spectrometry and analytical HPLC. After lyophilization, the TFA counterions were exchanged in 5% acetate (*v*/*v*).

The two nor-spermidine analogues (Nor-Leu and Nor-Phe) were synthesized through two-step reactions. In the first step of the reactions, the carboxylic acid group of Boc-protected amino acids (phenylalanine or leucine) was coupled with the two primary amine groups of the lipophilic norspermidine derivatives through an amide bond using HBTU as coupling agent. In the second step of the reaction, the final compounds were achieved through deprotection of Boc groups by using trifluoroacetic acid (TFA). Finally, the compounds were characterized by ^1^ H NMR and HR-MS, and their purity was determined by HPLC, which proved that the compounds were more than 95% pure.

Killer-FLIP Was Purchased from Biomatik (Kitchener, ON, Canada).

#### 4.1.2. Stock Solutions

Compounds’ stock solutions were prepared by dissolving each peptide/peptidomimetic in water. The UV spectrum was recorded, and the exact concentration was calculated through the absorbance at the wavelength corresponding to the maximum in the absorbance spectrum for each compound.

### 4.2. Instrumentation

Steady-state fluorescence experiments were carried out with a Fluoromax-4 fluorimeter (Horiba, Edison, NJ, USA). Time-resolved measurements were performed with a Lifespec-ps fluorometer (Edinburg instrument, Edinburgh, UK). Sample temperature was controlled to 25.0 °C (within 0.1 °C) with a thermostated water bath.

### 4.3. Liposome Preparation

Large unilamellar vesicles were prepared by dissolving in a 1:1 (*v*/*v*) chloroform/methanol mixture POPC/POPG lipids (2:1 molar ratio), and the appropriate fluorophore (Laurdan, NBD-PE, DPH, Pyr-PG, or Pyr-PC), or PEG-PE (2 mol% with respect to the total lipid concentration), when needed. The fluorophore was at a ratio of 1% with respect to the total lipids, except for Pyr-PG and Pyr-PC, which were 3%. Measurements with Killer-FLIP were conducted on POPS/POPC vesicles (1:1 molar ratio) to better mimic the composition of the cancer cell membrane. The solvents were evaporated under reduced argon atmosphere until a thin film was formed. Complete evaporation was ensured by applying a rotary vacuum pump for at least 2 h. The lipid film was hydrated with 10 mM phosphate buffer (pH 7.4) containing 140 mM NaCl and 0.1 mM EDTA. In leakage experiments, the lipid film was hydrated with 30 mM CF or calcein solution, titrated to pH 7.4 with NaOH, and containing 10 mM phosphate buffer and 80 mM NaCl to make it isotonic to dilution buffer. The liposome suspension was vigorously stirred and 10 freeze and thaw cycles were performed. The suspension was extruded 31 times through two stacked polycarbonate membranes with 100 nm pores, to ensure a narrow size distribution. Liposomes hydrated with the CF or Calcein solutions, respectively, were separated from unencapsulated dye by gel filtration on a Sephadex G-50 medium column. The final lipid concentration was determined by the Stewart method [81].

### 4.4. Liposome Leakage

Perturbation of membrane permeability was determined by measuring the fractional release of the fluorophore (Calcein or CF, respectively) entrapped inside liposomes at self-quenching concentration. This quantity can be measured directly by the increase in fluorescence intensity caused by the reduction in dye self-quenching 20 min after peptide addition. The fractional release was calculated as follows (Equation (1)):

(1) $$\mathrm{Released}\;\mathrm{fraction}\;=\;\frac{F-F_{0}}{F_{100}-F_{0}}$$

where *F*_0_ is the fluorescence of the probe before peptide addition, and *F*_100_ is the intensity after complete disruption of the vesicles caused by the addition of Triton X-100 (1 mM). The release kinetics were recorded for both probes with a FluoroMax-4 fluorimeter with excitation and emission wavelengths of 490 nm (bandwidth 1.5 nm) and 520 nm (bandwidth 0.2 nm), respectively.

### 4.5. Steady-State and Time-Resolved Anisotropy

All fluorescence measurements (steady-state and time-resolved anisotropy, generalized polarization, and lipid lateral mobility) were performed using a 1cm × 0.4 cm quartz cuvette, (Hellma, Müllheim, Germany) using an excitation path length of 0.4 cm.

Steady-state and time-resolved anisotropy were recorded by titrating a fixed liposome solution (lipid concentration 50 µM) with increasing concentrations of peptide/peptidomimetic. Perturbation of membrane dynamics caused by peptides or peptidomimetics was analyzed by experiments of steady-state and time-resolved anisotropy of three different probes, DPH, Laurdan and NBD-PE. In all cases, the probe to phospholipids molar ratio was fixed to 1:100.

For DPH steady-state anisotropy, the experimental conditions were set as follows: excitation wavelength 372 nm, emission wavelength 450 nm, bandwidth 4 nm and 385 nm cut-off filter. For NBD-PE, the excitation wavelength was set to 460 nm, emission 530 nm, bandwidth 4 nm and 495 nm cut-off filter.

For Laurdan, the excitation wavelength was set to 372 nm, emission to 475 nm, cut-off filter 385 nm and bandwidth 3, 5 nm in excitation and emission, respectively.

Each experimental point was determined fifteen times, and the mean value was reported.

Time-resolved fluorescence measurements were performed on a Lifespec-ps setup equipped with automatic Glan–Thompson polarizers, a cooled micro-channel plate detector and working in the time correlated single photon counting mode (TCSPC) (Edinburgh Instruments, Livingston, UK). The excitation sources were a diode laser at 440 nm (NBD-PE) and 372 nm (DPH and Laurdan), emission band pass was fixed at 8 nm, and emission wavelength 450 nm (DPH), 530 nm (NBD-PE), 475 nm (Laurdan).

Data were fitted with a biexponential decay, according to the following Equation (2):

(2) $$r\left(t\right)=r_{0}\left[f_{1}e^{\frac{-t}{\phi_{1}}}+f_{2}e^{\frac{-t}{\phi_{2}}}+f_{\infty }\right]$$

where *r*_0_ is the limiting anisotropy, *ϕ* the rotational correlation times and *f* the fraction of residual anisotropy. The average rotational correlation time has been calculated using the following Equation (3):

(3) $$\phi \geq \frac{f_{1}\phi_{1}+f_{2}\phi_{2}}{f_{1}+f_{2}}$$

### 4.6. Lipid Lateral Mobility

Liposomes were labelled with the fluorescent probe pyrene covalently linked to the hydrocarbon chain of phospholipids. Liposomes with two different compositions were prepared: POPC/POPG/Pyr-PG (66:30:3 molar ratio) and POPC/POPG/Pyr-PC (63:33:3 molar ratio).

Pyrene fluorescence spectra (370–700 nm) were recorded by titrating a fixed liposome solution (lipid concentration 50 µM) with increasing concentrations of peptide (excitation wavelength 328 nm, integration time 0.1 s, excitation and emission bandwidths 1 nm and 1.5 nm respectively). Excimers over monomer fluorescence intensity was measured as the ratio between fluorescence values at 475 nm and 397 nm.

### 4.7. Generalized Polarization

Spectra of Laurdan-labelled liposomes were recorded from 400 nm to 520 nm by titrating a fixed liposome solution (lipid concentration 50 µM) with increasing concentrations of peptide (excitation wavelength 372 nm, 2 nm bandwidth both in excitation and emission). The generalized polarization was calculated as follows (Equation (4)):

(4) $$GP=\frac{I_{435}-I_{500}}{I_{435}+I_{500}}$$

where *I*_λ_ is the fluorescence intensity relating to a specific emission wavelength λ.

### 4.8. Turbidity Measurements

Peptide-induced vesicle aggregation was followed by titrating a fixed lipid concentration (50 μM) with increasing concentrations of the membrane-active molecule. The variation of the optical density at 600 nm was recorded using a Cary 100 Scan UV-visible spectrophotometer (Agilent, Santa Clara, CA, USA). Each experimental point was determined three times, and the mean value was reported.

### 4.9. Dynamic Light Scattering Measurements

Liposome size was determined by dynamic light scattering (DLS) using a Malvern Zetasizer (Malvern, UK) equipped with a 633 nm He–Ne laser operating in backscatter mode (173°). Samples (70 μL) were analyzed in disposable microcuvettes after equilibration for 60 s at 25 °C. Three consecutive measurements were acquired for each sample, and the results were averaged. Water was used as the dispersant (refractive index = 1.330; viscosity = 0.8872 cP at 25 °C), while the refractive index and absorption values assigned to the liposomes were 1.45 and 0.001, respectively. The measurement position was set to 3 mm and the attenuator to 10. Liposome size was measured before and after the addition of the maximum concentration tested of each compound (lipid concentration: 50 μM).

### 4.10. Comparison Between Membrane-Active Peptides and Peptidomimetics

The surface area occupied by the membrane-active compounds investigated in this study was assumed to be similar across all peptides, given their comparable charge, sequence length, and secondary structure. More in detail, we started from the average value measured in the simulations of PMAP-23 bound to the membrane (5.4 nm^2^) [11], and, to account for minor differences in peptide length, a normalization factor was applied by multiplying the area by the ratio of the number of residues in the specific peptide analyzed to 23 (the number of residues for PMAP-23). Regarding the peptidomimetics, the surface areas of Nor-Leu and Nor-Phe were assumed to be equal to that of a non-spermidine analogue (Nor-Trp—0.8 nm^2^), for which it was also calculated as an average in MD simulations when bound to bilayers [33].

### 4.11. Software

Graphs were generated using KaleidaGraph 5.0 (Synergy Software, Reading, PA, USA). The graphical abstract was prepared with the assistance of ChatGPT (OpenAI, https://www.chatgpt.com).

## 5. Conclusions

All the parameters examined in this study, membrane viscosity, hydration, and lipid lateral mobility, consistently point in the same direction, indicating that membrane-active molecules cause a global decrease in the membrane mobility on the nanosecond timescale, regardless of their composition, length, charge, the depth of each used probe within the bilayer, or mechanism of action in vesicles.

While membrane permeabilization is achieved at relatively low peptide concentrations, alterations in membrane dynamics become evident only at higher concentrations, most likely reflecting an almost complete coverage of the bilayer surface, where alternative peptide-lipid arrangements may take place.

When normalized for molecular size and surface area, peptidomimetics show the same activity range as AMPs, confirming that near-complete membrane coverage is required to induce perturbation and bilayer stiffening.

The workflow designed here, transferable to bacteria, too [13], could help to distinguish among membrane-active compounds for which the “Sand-in-gearbox” mechanism could play a role.

## Figures and Tables

**Table 1:** Amino-acidic sequence, net charge and normalization factor of the investigated peptides.

Peptide	Sequence ^1^	Net Charge at pH 7.4	Estimated Surface Area ^2^
*Magainin 2*	GIGKFLHSAKKFGKAFVGEIMNS	+3	5.4
*Melittin*	GIGAVLKVLTTGLPALISWIKRKRQQ-NH_2_	+5	6.1
*LAH4*	KKALLALALHHLAHLALHLALALKKA-NH_2_	+4	6.1
*Killer-FLIP*	GRKKRRQRRRFFWSLCTA	+8	4.2

^1^ Hydrophobic, basic, His, and other residues are reported in green, blue, orange and black, respectively. ^2^ The values are used to normalize the surface area occupied by the membrane-active compounds. See Section 4.10 for the details.

## Data Availability

Data can be furnished by authors upon request.

## Supplementary Materials

The following supporting information can be downloaded at: https://www.mdpi.com/article/10.3390/antibiotics15080741/s1, Figure S1: Comparison of the dye leakage induced by LAH4 at pH7.4 from POPC/POPG (2:1 molar ratio) liposomes (lipid concentration 50 μM), 20 min after peptide addition, in the case of calcein and CF; Figure S2: Membrane-perturbing activity of membrane-active compound killer-FLIP from POPC/POPS (1:1 molar ratio) liposomes. Adapted from [32]; Figure S3: Fraction of CF leakage induced by Nor-Leu (continuous red line) and Nor-Phe (dashed red line) from POPC/POPG (2:1 molar ratio) liposomes, (lipid concentration 50 μM) 20 min after peptidomimetics addition; Figure S4: Steady-state anisotropy of Laurdan as a function of melittin concentration. Membrane composition: POPC/POPG 2:1 molar ratio, 1% probe (red circle) or 0.1% (orange circle). Lipid concentration 50 μM. Λ_exc_ = 372 nm, λ_em_ = 475 nm. Bandwidth = 2 nm, 4 nm for excitation and emission, respectively. The concentration range in which the peptide-induced membrane leakage goes from 3% to 97% is reported as a shaded area, for comparison; Figure S5: Kinetic curves of carboxyfluorescein (CF) release from liposomes after the addition of increasing concentration of magainin 2 (A), melittin (B), LAH4 pH 5 (C), LAH4 pH 7.4 (D), Nor-Leu (E), Nor-Phe (F), and Killer-FLIP (G). Liposome composition: POPC/POPG 2:1 except for killer-FLIP, for which a membrane composition of POPC/POPS 1:1 was selected; Figure S6: Size distribution of liposomes alone (panel A) and after the addition of peptides/peptidomimetics obtained by light scattering measurements. B: Magainin 2 30 μM; C: Melittin 30 μM; D: LAH4, pH 7.4, 10 μM; E: Nor-Phe 50 μM; F: Nor-Leu 50 μM; G: Killer-FLIP 8 μM. All the measures were carried out on POPC/POPG 2:1 molar ratio vesicles; Figure S7: Vesicle agglutination. (A) Turbidity measurements on liposome suspension (vesicle composition: POPC/POPG 2:1 molar ratio, lipid concentration 50 μΜ) with and without peptidomimetics. (A): red Nor-Leu, blue Nor-Phe). (B) Optical density at 600 nm of vesicles (lipid concentration = 50 μΜ) in the presence of PEG-PE (empty circles) and without it (full circles), titrated with increasing melittin concentrations. (C) DPH anisotropy inserted into liposomes with PEG-PE, (POPC, POPG, PEG-PE, DPH; 66, 31, 2, 1 mole %, respectively) resulting from titration with increasing concentrations of melittin. Lines are represented only as a guide for the eye. Figure S8: Changes in the fluorescence spectra of Laurdan in POPC/POPG vesicles (lipid concentration = 50 μM, 2:1 phospholipids molar ratio, probe 1%) upon addition of melittin. Λ_exc_ = 372 nm, bandwidth = 3 nm, and λ_em_ = 475 nm, bandwidth = 4 nm. Table S1: Parameters of a biexponential fit of time-resolved anisotropy for magainin 2, melittin and LAH4 with the different probs; Table S2: Membrane coverage [82].

## Abbreviations

The following abbreviations are used in this manuscript:

AMR	Antimicrobial resistance
AMP	Antimicrobial peptide
CF	carboxyfluorescein
DDQ	Depth-depending quenching
MD	molecular dynamics
GP	generalized polarization
