# Supplementary material for: Bilayer Permeabilization and the ‘Sand-in-a-Gearbox’ Mechanism for Membrane-Active Molecules: Which Is Which?
Source: Antibiotics (Basel). 2026 Jul 31;15(8):741. doi: 10.3390/antibiotics15080741 (PMC13509787; doi:10.3390/antibiotics15080741)
Supplement: Supplementary file 1 [file antibiotics-15-00741-s001.zip › antibiotics-4376988-supplementary.pdf]

SUPPORTING INFOTMATION

Bilayer Permeabilization and the 'Sand-in-a-Gearbox' Mechanism for Membrane-Active Molecules: Which Is Which?

Zahra Vaezi [1,2], Annalisa Bortolotti [1], Cristiano Di Stefano [1], Simone Bonacorsi [1], Valerio Santucci [1], Federico Carneri [1,3], Christopher Aisenbrey [4], Mohini M. Konai [5,6], Yash Acharya [5], Mariano Venanzi [1], Roya Khosravi-Far [7], Burkhard Bechinger [4,8], Jayanta Haldar [5], Lorenzo Stella [1], Daniela Roversi [1,*] and Gianfranco Bocchinfuso [1,*]

[1] Department of Chemical Sciences and Technologies, Tor Vergata University of Rome, Via della Ricerca Scientifica 1, 00133 Rome, Italy; zahra.vaezi@modares.ac.ir (Z.V.); bortolottiannalisa@libero.it (A.B.); cristianodst633@gmail.com (C.D.S.); simone.bonacorsi92@gmail.com (S.B.); valerio135v@gmail.com (V.S.); federico.carneri@uniroma2.it (F.C.); venanzi@uniroma2.it (M.V.); stella@stc.uniroma2.it (L.S.)

[2] Department of Biomaterial, Faculty of Interdisciplinary Science and Technologies, Tarbiat Modares University, Tehran, 14115-154, Iran

[3] Photoinduced Processes and Technologies Doctoral School, Department of Chemistry, Biology and Biotechnology, Perugia University, 06123 Perugia, Italy

[4] RMN et Biophysique des Membranes, Institut de Chimie de Strasbourg, UMR 7177, Université de Strasbourg, 4, Rue Blaise Pascal, 67000 Strasbourg, France; aisenbrey@unistra.fr (C.A.); bechinge@unistra.fr (B.B.)

[5] Jawaharlal Nehru Centre for Advanced Scientific Research, Bengaluru 560064, India; mmkonai@tezu.ernet.in (M.M.K.); yash@jncasr.ac.in (Y.A.); jayanta@jncasr.ac.in (J.H.)

[6] Medicinal Chemistry and Biomaterial Laboratory (MCBL), Department of Chemical Sciences, Tezpur University, Napaam, Tezpur, Assam 784028, India

[7] InnoTech Precision Medicine, Boston, MA 02142, USA; roya@innotechprecisionmed.com

[8] Institut Universitaire de France, 75005 Paris, France

[*] Correspondence: daniela.roversi@uniroma2.it (D.R.); gianfranco.bocchinfuso@uniroma2.it (G.B.)

[†] The authors dedicate this article to the memory of Lorenzo Stella, who passed away on 24 August 2025, and whose inspiration made it possible.

Table S1. Parameters of a biexponential fit of time-resolved anisotropy for magainin 2, melittin, and LAH4 with the different probs

	**[Magainin 2] 0.0 µM**	**[Magainin 2] 19 µM**	**[Melittin] 0.0 µM**	**[Melittin] 1.0 µM**	**[LAH4] 0.0 µM**	**[LAH4] 10.0 µM**	**[LAH4] 0.0 µM**	**[LAH4] 10.0 µM**
**DPH**					pH= 5	pH= 5	pH= 7.4	pH= 7.4
	**τ_1= 8.89<br>τ_2= 2.5<br>$<\tau>$= 7.82**	**τ_1= 9.22<br>τ_2= 2.58<br>$<\tau>$= 7.93**	**τ_1= 8.9<br>τ_2= 2.42<br>$<\tau>$= 7.58**	**τ_1= 9.12<br>τ_2= 2.16<br>$<\tau>$= 7.84**	**τ_1= 9.09<br>τ_2= 2.57<br>$<\tau>$= 7.24**	**τ_1=8.9<br>τ_2=2.64<br>$<\tau>$=6.74**	**τ_1=9.39<br>τ_2=2.86<br>$<\tau>$=7.91**	**τ_1=10.21<br>τ_2=3.11<br>$<\tau>$=7.90**
ϕ_1 (ns)	3.8±0.3	4.6±0.4	3.2±0.1	4.4±0.3	3.2±0.2	6.0±0.6	3.4±0.1	7.5±0.6
f_1	0.46±0.05	0.44±0.03	0.58±0.03	0.46±0.04	0.57±0.04	0.45±0.04	0.58±0.02	0.42±0.02
ϕ_2 (ns)	1.0±0.1	0.9±0.1	0.68±0.08	1.10±0.10	0.8±0.1	1.4±0.2	0.63±0.06	1.1±0.1
f_2	0.44±0.05	0.42±0.03	0.32±0.03	0.40±0.04	0.31±0.04	0.34±0.04	0.32±0.02	0.35±0.02
$<\phi>$	2.4±0.5	2.8±0.5	2.3±0.2	2.9±0.4	2.3±0.3	4.0±0.8	2.4±0.2	4.6±0.8
f_∞	0.10±0.05	0.14±0.03	0.10±0.03	0.14±0.04	0.12±0.04	0.21±0.04	0.07±0.02	0.23±0.02
χ^2_{rid}	1.04	1.03	0.9	1.0	1.0	0.9	1.0	1.0
**NBD**					pH= 5	pH= 5	pH= 7.4	pH= 7.4
	**τ_1= 8.25<br>τ_2= 1.32<br>$<\tau>$= 5.94**	**τ_1= 8.21<br>τ_2= 1.43<br>$<\tau>$= 5.94**	**τ_1= 8.38<br>τ_2= 1.54<br>$<\tau>$= 6.10**	**τ_1= 8.43<br>τ_2= 1.66<br>$<\tau>$= 6.18**	**τ_1=8.53<br>τ_2=1.5<br>$<\tau>$=5.21**	**τ_1=8.53<br>τ_2=1.76<br>$<\tau>$=4.55**	**τ_1=8.81<br>τ_2=1.62<br>$<\tau>$=5.54**	**τ_1=8.79<br>τ_2=1.74<br>$<\tau>$=4.5**
ϕ_1 (ns)	3.8±0.2	4.0±0.3	3.1±0.1	4.3±0.2	3.8±0.1	6.2±0.3	4.3±0.1	7.3±0.5
f_1	0.46±0.02	0.47±0.03	0.54±0.01	0.42±0.02	0.47±0.01	0.36±0.01	0.50±0.01	0.32±0.01
ϕ_2 (ns)	0.59±0.05	0.76±0.06	0.32±0.03	0.67±0.05	0.22±0.02	0.16±0.01	0.29±0.04	0.20±0.02
f_2	0.48±0.02	0.4±0.02	0.39±0.01	0.46±0.02	0.38±0.01	0.38±0.01	0.33±0.01	0.30±0.01
$<\phi>$	2.2±0.3	2.5±0.4	1.9±0.1	2.4±0.3	2.2±0.2	3.1±0.4	2.7±0.2	3.9±0.5
f_∞	0.06±0.02	0.13±0.02	0.07±0.01	0.12±0.02	0.15±0.01	0.26±0.01	0.17±0.01	0.38±0.01
χ^2_{rid}	0.85	0.91	0.8	0.9	0.8	1.0	1.0	1.0
**Laurdan**								
	**τ_1= 4.02<br>τ_2= 1.11<br>$<\tau>$= 2.86**	**τ_1= 4.09<br>τ_2= 1.33<br>$<\tau>$= 3.10**	**τ_1= 3.9<br>τ_2= 1.22<br>$<\tau>$= 2.88**	**τ_1= 3.4<br>τ_2= 1.17<br>$<\tau>$= 2.96**				
ϕ_1 (ns)	3.0±0.1	4.1±0.3	3.3±0.1	3.9±0.4				
f_1	0.61±0.01	0.57±0.03	0.58±0.02	0.48±0.05				
ϕ_2 (ns)	0.45±0.05	0.90±0.10	0.59±0.06	1.20±0.20				
f_2	0.34±0.01	0.32±0.03	0.33±0.02	0.34±0.05				
$<\phi>$	2.1±0.1	3.0±0.4	2.3±0.2	2.8±0.4				
f_∞	0.05±0.01	0.11±0.03	0.09±0.02	0.18±0.07				
χ^2_{rid}	0.8	1.2	0.9	1.0				

Table S2: Membrane coverage

Membrane-active molecule	Molecular footprint (nm^2)	Lipid molecular area (nm^2)*	Compound/lipid ratio for total membrane coverage**	Experimental membrane coverage (P/L)
Magainin 2	5.4	0.63	0.25	0.6 (30/50)
Melittin	6.1	0.63	0.2	0.6 (30/50)
LAH4	6.1	0.63	0.2	0.2 (10/50)
Killer-FLIP	4.2	0.59	0.3	0.2 (10/50)
Nor-Leu	0.8	0.63	1.7	1 (50/50)
Nor-Phe	0.8	0.63	1.7	0.6 (30/50)

* Lipid molecular area was taken from ref. 82 in the Main Text, and the weighted average based on each membrane composition was considered.

** The compound/lipid ratio was divided by a factor of two to consider the worst scenario, in which membrane permeabilization occurs, and the compound interacts with both the external and internal leaflets of the liposome membrane

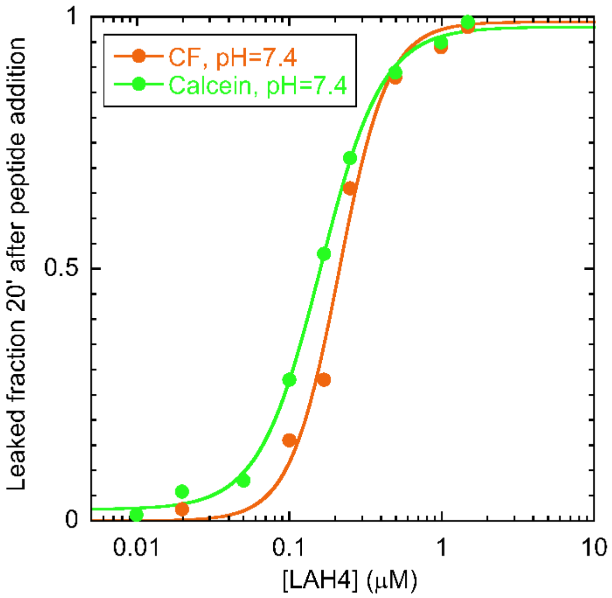

Figure S1. Comparison of the dye leakage induced by LAH4 at pH7.4 from POPC/POPG (2:1 molar ratio) liposomes (lipid concentration 50 μM), 20 minutes after peptide addition, in the case of calcein and CF

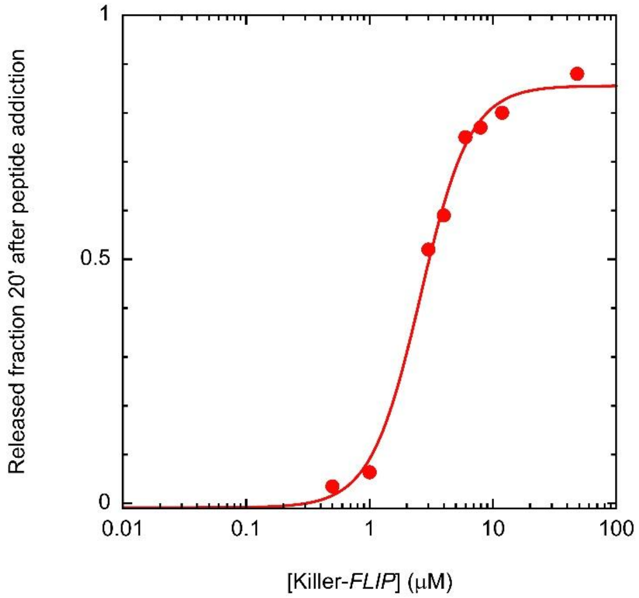

Figure S2. Membrane-perturbing activity of membrane-active compound killer-FLIP from POPC/POPS (1:1 molar ratio) liposomes. Adapted from [32].

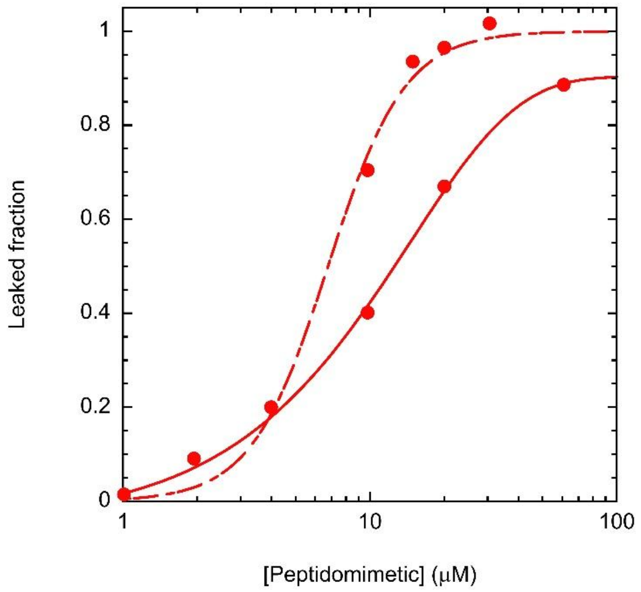

Figure S3. Fraction of CF leakage induced by Nor-Leu (continuous red line) and Nor-Phe (dashed red line) from POPC/POPG (2:1 molar ratio) liposomes, (lipid concentration 50 μM) 20 minutes after peptidomimetics addition.

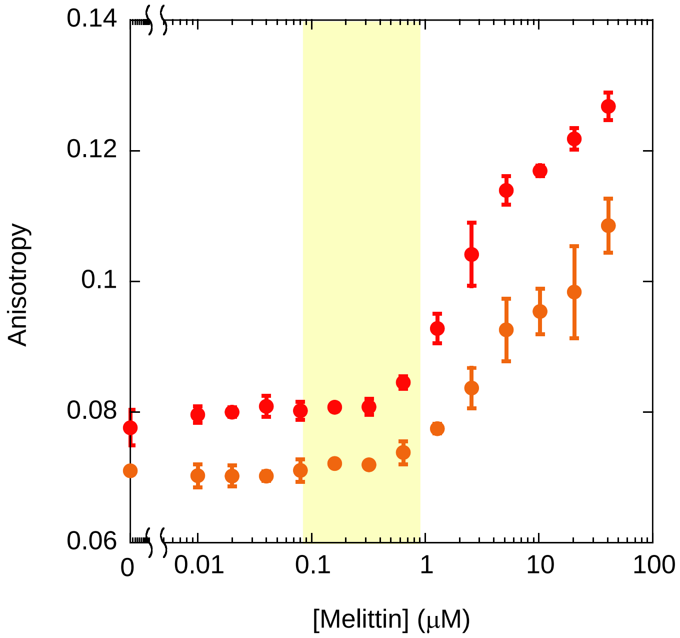

Figure S4. Steady-state anisotropy of Laurdan as a function of melittin concentration. Membrane composition: POPC/POPG 2:1 molar ratio, 1% probe (red circle) or 0.1% (orange circle). Lipid concentration 50 μM. λexc= 372 nm, λem= 475 nm. Bandwidth=2 nm, 4nm for excitation and emission, respectively. The concentration range in which the peptide-induced membrane leakage goes from 3% to 97% is reported as a shaded area, for comparison.

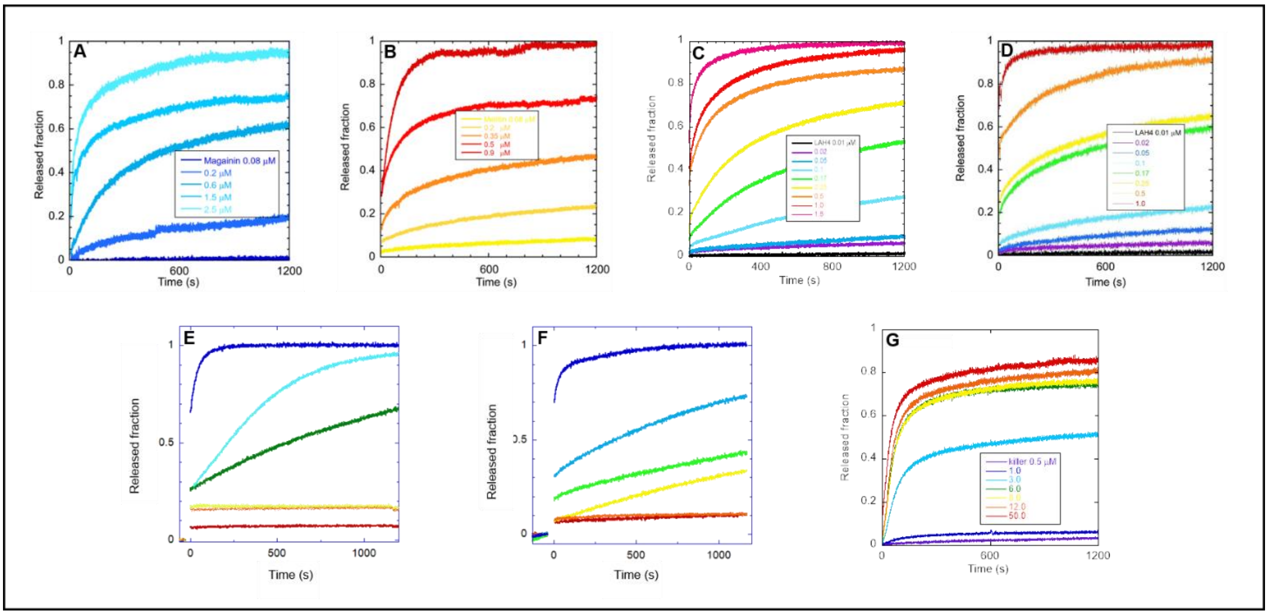

Figure S5: Kinetic curves of carboxyfluorescein (CF) release from liposomes after the addition of increasing concentration of magainin 2 (A), melittin (B), LAH4 pH 5 (C), LAH4 pH 7.4 (D), Nor-Leu (E), Nor-Phe (F), and Killer-FLIP(G). Liposome composition: POPC/POPG 2:1 except for killer-FLIP, for which a membrane composition of POPC/POPS 1:1 was selected.

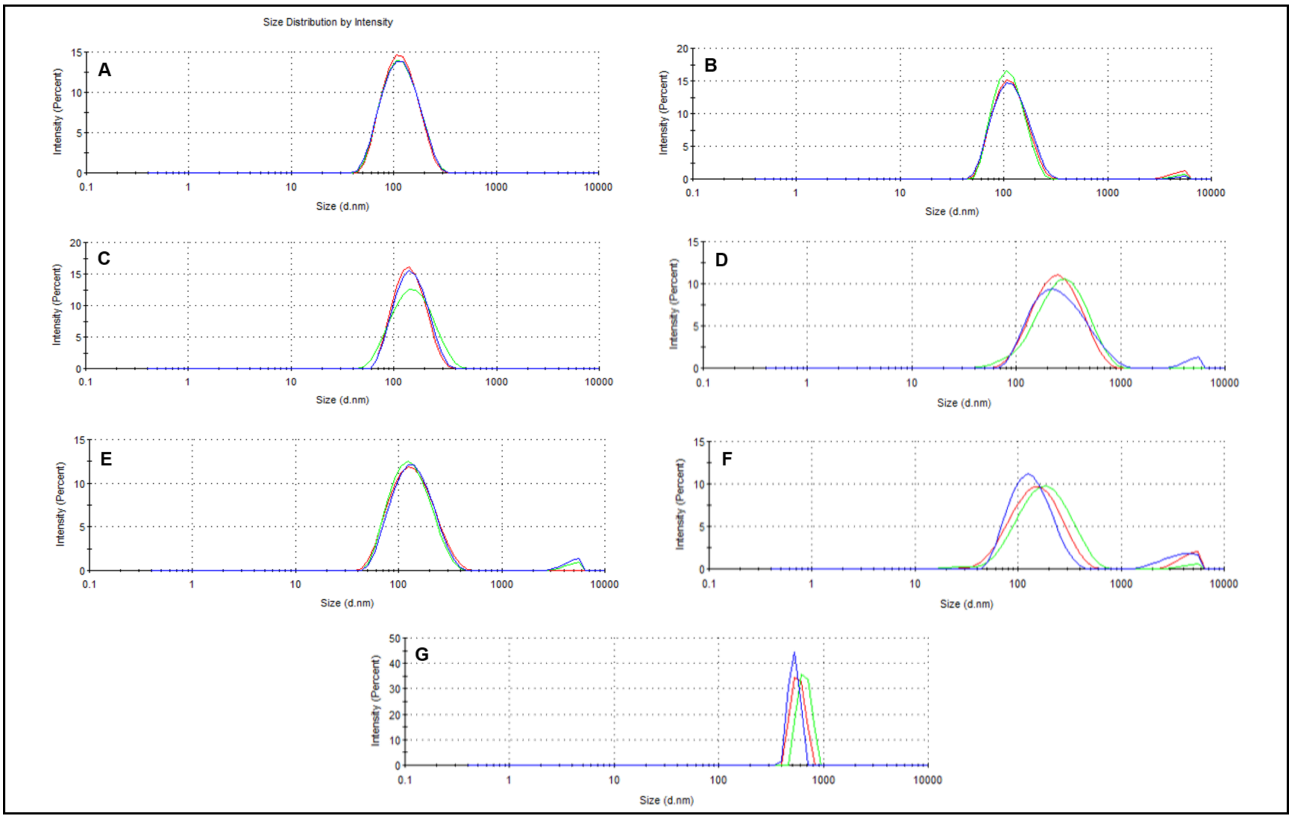

Figure S6: Size distribution of liposomes alone (panel A) and after the addition of peptides/peptidomimetics obtained by light scattering measurements. B: Magainin 2 30 μM; C: Melittin 30 μM; D: LAH4, pH 7.4, 10 μM; E: Nor-Phe 50 μM; F: Nor-Leu 50 μM; G: Killer-FLIP 8 μM. All the measures were carried out on POPC/POPG 2:1 molar ratio vesicles

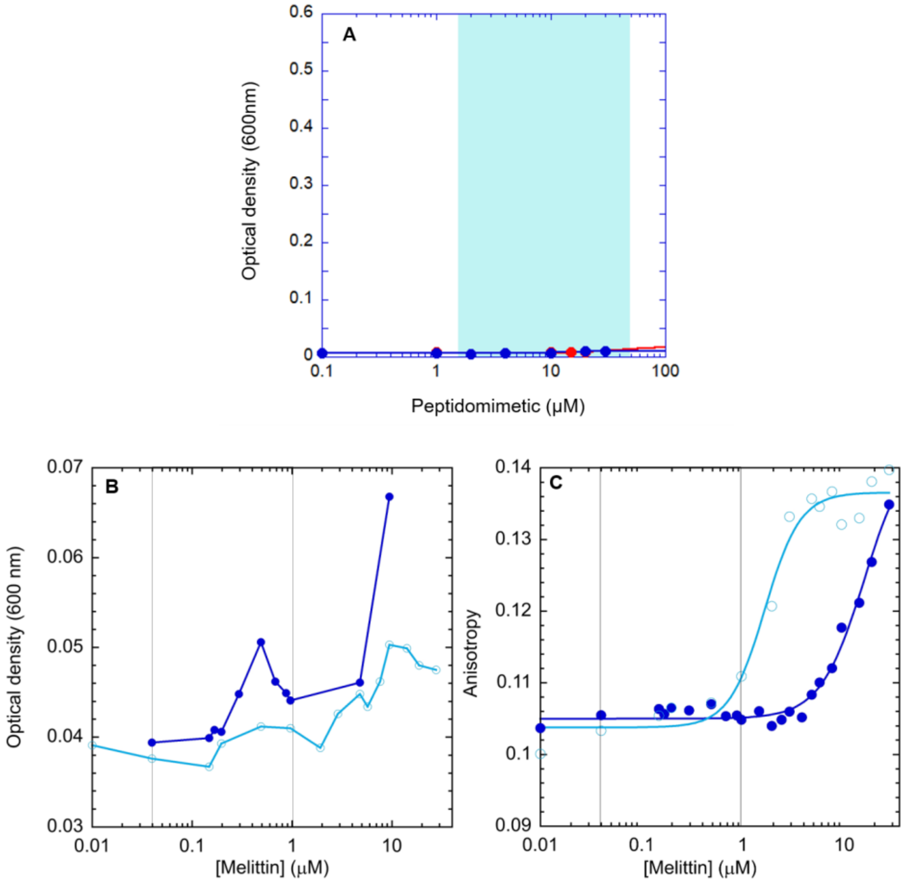

Figure S7: Vesicle agglutination. A) Turbidity measurements on liposome suspension (vesicle composition: POPC/POPG 2:1 molar ratio, lipid concentration 50 μM) with and without peptidomimetics. A): red Nor-Leu, blue Nor-Phe). B) Optical density at 600 nm of vesicles (lipid concentration = 50 μM) in the presence of PEG-PE (empty circles) and without it (full circles), titrated with increasing melittin concentrations. C) DPH anisotropy inserted into liposomes with PEG-PE, (POPC, POPG, PEG-PE, DPH; 66, 31, 2, 1 mole %, respectively) resulting from titration with increasing concentrations of melittin. Lines are represented only as a guide for the eye.

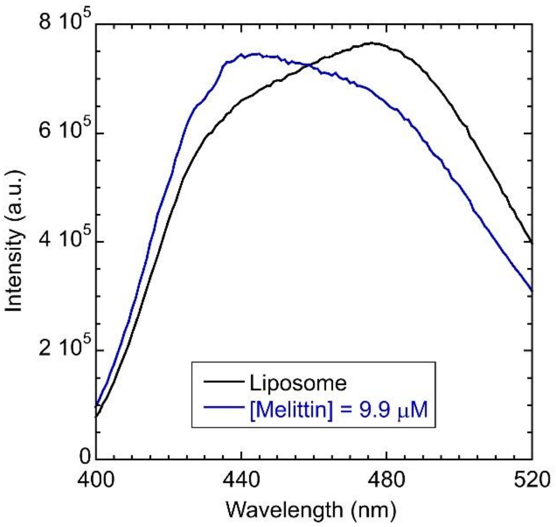

Figure S8: Changes in the fluorescence spectra of Laurdan in POPC/POPG vesicles (lipid concentration = 50 μM, 2:1 phospholipids molar ratio, probe 1%) upon addition of melittin. λexc= 372 nm, bandwidth= 3 nm, and λem= 475 nm, bandwidth= 4 nm
